# Hybrid Multilayer Epoxy Composites Filled with Mixed-Phase Bismuth- and Tungsten-Containing Nanoparticles for Lead-Free ^99m^Tc Shielding in Occupational Nuclear Medicine

**DOI:** 10.3390/polym18192434

**Published:** 2026-10-06

**Authors:** Suphalak Khamruang Marshall, Wuttipat Wattanaphonpinich

**Affiliations:** Department of Radiology, Faculty of Medicine, Prince of Songkla University, Songkhla 90110, Thailand

**Keywords:** technetium-99m, gamma-ray shielding, Bi_2_O_3_/WO_3_ nanoparticles, lead-free epoxy nanocomposites, multilayer shielding, occupational radiation protection

## Abstract

Lead-free polymer composites are being developed to reduce occupational radiation exposure associated with nuclear medicine procedures. This study fabricated monolithic and four-layer hybrid Bi_2_O_3_/WO_3_ nanoparticle-filled diglycidyl ether of bisphenol A epoxy composites for attenuating the 140.5-keV photons emitted by technetium-99m (^99m^Tc). Bi- and W-containing powders were prepared by wet-chemical precipitation and incorporated into the epoxy matrix at 25 and 50 parts per hundred parts of resin. Alternating B50/W50/B50/W50 (ML-BW50) and reverse-layer configurations were produced by sequential casting with a total specimen thickness of 2.0 cm. The nanoparticle dispersions and cured composites were evaluated using DLS, zeta-potential analysis, FE-SEM–EDX, XRD, FTIR spectroscopy, PBS contact-angle measurements, and supplementary tensile testing. Shielding performance was measured at ^99m^Tc activities of 925 MBq (25 mCi) and 6.105 GBq (165 mCi) using an anthropomorphic thorax phantom and an electronic personal dosimeter under a fixed source–shield–detector geometry. The Bi_2_O_3_ and WO_3_ dispersions exhibited Z-average hydrodynamic diameters of 646.41 and 413.38 nm and zeta potentials of −33.70 and −31.65 mV, respectively. FE-SEM and EDX analyses documented compositionally differentiated Bi-rich and W-rich layers in ML-BW50. XRD identified a mixed-phase Bi- and W-containing filler system, including α-Bi_2_O_3_, bismuth oxide carbonate hydroxide, WO_3_, and residual Na_2_WO_4_·2H_2_O, while FTIR spectra retained the characteristic bands of the cured epoxy network. ML-BW50 exhibited a maximum tensile stress of 4.008 MPa, a terminal strain of 74.294%, and a tensile toughness of 2.250 MJ·m^−3^, representing the highest tensile toughness among the investigated epoxy-based formulations. The shielding formulation significantly affected the background-corrected personal dose equivalent, Hp(10), at both activities (both *p* < 0.0001), and the multilayer specimens reduced Hp(10) by approximately 45–72% relative to their corresponding monolithic composites. ML-BW50 was the most effective lead-free formulation, producing Hp(10) values of 33.15 ± 2.75 and 228.42 ± 6.50 µSv and radiation-shielding efficiencies of 77.42% and 82.70% at 925 MBq and 6.105 GBq, respectively. ML-BW50 and the 0.5-mm lead reference produced statistically comparable Hp(10) values at 925 MBq, whereas ML-BW50 produced a 9.12% lower Hp(10) than lead at 6.105 GBq. For the same 10 × 10 cm projected area, ML-BW50 had a mass of 44.0 g compared with 77.0 g for lead, representing a 42.9% mass reduction, and exhibited a mean PBS contact angle of approximately 102.3°. Collectively, ML-BW50 combined effective ^99m^Tc attenuation, reduced specimen mass, differentiated multilayer organization, low PBS wettability, and favorable tensile-energy absorption, supporting its continued development as a lead-free occupational shielding material for nuclear medicine.

## 1. Introduction

Technetium-99m (^99m^Tc) remains the principal diagnostic radionuclide used in conventional nuclear medicine because its approximately 6 h physical half-life, predominant 140.5 keV photon emission, and favorable radiolabeling chemistry permit clinically useful imaging with comparatively low patient radiation burden. Nevertheless, the preparation, dispensing, administration, and imaging of ^99m^Tc radiopharmaceuticals create recurrent external-exposure scenarios for radiopharmacists, nuclear medicine technologists, nurses, and other personnel working near radioactive sources or injected patients [1,2,3,4]. Although individual occupational doses are generally maintained below regulatory limits, compliance with a dose limit alone does not constitute optimization. Repeated low-dose exposure, nonuniform workflow-related dose distributions, and potentially elevated doses near generators, dispensing stations, syringes, and recently administered patients support continued application of the principles of time, distance, and shielding [5]. Consequently, there is a practical need for shielding materials that are effective specifically at the 140.5 keV photon energy of ^99m^Tc, and that can be fabricated into compact barriers, source containers, work-surface panels, syringe-handling devices, and other application-specific components.

Photon-shielding performance is strongly energy-dependent and cannot be inferred solely from material density or nominal filler concentration. For a homogeneous absorber under ideal narrow-beam conditions, photon transmission is conventionally described by $I\;=\;I_{0}e^{-\mu x}$ where $I_{0}$ and I are the incident and transmitted photon intensities, respectively, x  is the absorber thickness, and μ is the linear attenuation coefficient (LAC). The mass attenuation coefficient (MAC), $\mu_{\rho }$, separates the influence of density from the elemental photon-interaction probability and can be estimated for compounds or mixtures using elemental cross-section databases such as NIST XCOM [6,7]. At 140.5 keV, photon attenuation occurs primarily through photoelectric absorption and incoherent (Compton) scattering. Compton scattering is related mainly to electron density, whereas the photoelectric contribution increases strongly with atomic number, approximately as $\frac{Z^{n}}{E^{m}}\;$ away from absorption edges, where the exponents depend on photon energy and elemental composition [7,8,9]. The low-Z constituents of unmodified polymers therefore provide comparatively weak photoelectric attenuation, whereas high-Z fillers can markedly increase photon-removal probability.

Lead has traditionally been used for photon shielding because of its high atomic number (Z = 82), high density, availability, and effective attenuation over a broad energy range. Nevertheless, conventional lead shielding presents several disadvantages, including toxicity, environmental persistence, difficult end-of-life disposal, substantial weight, limited flexibility, and potential occupational contamination during fabrication or degradation [10]. These limitations have stimulated the development of lead-free shielding materials incorporating high-atomic-number elements and metal oxides into polymeric, elastomeric, ceramic, glass, and cementitious matrices. Among these approaches, polymer-based composites are particularly attractive because they can be fabricated at relatively low temperatures and shaped into lightweight panels, coatings, barriers, syringe shields, and wearable protective components [10,11,12].

Accordingly, polymer matrices offer several processing advantages for alternative radiation-shielding systems, including low-temperature fabrication, corrosion resistance, geometric adaptability, and the capacity to immobilize high-Z particles within a continuous phase. Epoxy resin is particularly attractive because of its strong adhesion, low curing shrinkage, dimensional stability, chemical resistance, and compatibility with diverse inorganic fillers. However, neat epoxy consists predominantly of low-atomic-number elements and consequently provides limited intrinsic attenuation at diagnostic photon energies. Its shielding performance can be improved by incorporating high-Z metal-oxide fillers, which increase composite density and photon-interaction probability. Previous studies have demonstrated enhanced attenuation using WO_3_, Bi_2_O_3_, MgO, BaO, HfO_2_, and related oxides in epoxy and other polymer matrices [13,14]. These improvements are accompanied by important processing and mechanical trade-offs. Increasing the heavy-oxide content generally enhances linear attenuation but also raises the viscosity of the uncured resin, complicates particle wetting and dispersion, promotes agglomeration and air entrapment, and may permit density-driven settling before gelation. The resulting interfacial defects and heterogeneity can reduce strain at break, fracture resistance, and overall structural reliability [12,15,16]. Radiation-shielding performance should therefore be evaluated together with filler dispersion, porosity, interfacial bonding, density, and mechanical behavior. A composite exhibiting favorable theoretical attenuation but severe agglomeration, brittleness, or structural defects may ultimately be unsuitable for practical occupational shielding.

Thus, bismuth(III) oxide (Bi_2_O_3_) is a prominent lead-free filler because bismuth has a high atomic number (Z = 83), while Bi_2_O_3_ combines favorable photon attenuation with lower toxicity than conventional lead compounds. Previous investigations have shown that incorporating Bi_2_O_3_ into epoxy, polymethyl methacrylate, silicone, and related polymers increases the linear attenuation coefficient and reduces the half-value layer, particularly within low- and intermediate-energy photon fields [15,17]. Additionally, Muthamma et al. demonstrated that Bi_2_O_3_ particle size and loading affected both gamma-ray attenuation and the thermal and mechanical behavior of epoxy composites [17]. Furthermore, Hedaya et al. likewise reported differences between micro- and nanoscale Bi_2_O_3_–epoxy systems, but their results also illustrated the importance of composite density and particle distribution when interpreting particle-size effects [18]. Other studies have used mineral phases, graphene-derived structures, or surface-modified fillers to reduce agglomeration and improve the interaction between Bi_2_O_3_ and polymer matrices [11,13,19].

Furthermore, tungsten (VI) oxide (WO_3_) is also attractive because tungsten has a high atomic number (Z = 74), while WO_3_ offers chemical stability, relatively high density, and compatibility with several polymer-processing routes. WO_3_-containing epoxy and elastomer composites have demonstrated improved attenuation over a wide photon-energy range [13,15,20]. Molina Higgins et al. reported that incorporating WO_3_ nanoparticles increased the mass attenuation coefficient of epoxy composites, with the largest improvement occurring at lower photon energies [16]. Verdipoor et al. compared WO_3_, Bi_2_O_3_, and PbO particles in a polymer matrix and showed that shielding performance depended on both filler chemistry and particle scale [14]. More recently, silicon–epoxy composites containing mixed micro- and nanoscale WO_3_ displayed stronger attenuation than formulations containing only microscale WO_3_, although the improvement was also associated with differences in density and packing efficiency [13]. Direct experimental evaluation of WO_3_-reinforced polyisoprene against ^99m^Tc further demonstrated the potential of tungsten-based polymer composites for nuclear medicine shielding [21].

Correspondingly, a hybrid Bi_2_O_3_/WO_3_ system is scientifically interesting because the two oxides differ in atomic number, density, K-edge position, particle morphology, and interfacial behavior. The K-absorption edges of tungsten and bismuth occur at approximately 69.5 and 90.5 keV, respectively, below the 140.5 keV primary photon energy of ^99m^Tc [7]. Both fillers can therefore contribute substantially to photoelectric absorption at this energy. In addition, photoelectric interactions can generate characteristic fluorescence photons; whether these secondary photons escape from or are reabsorbed within the composite depends on composition, thickness, particle distribution, and irradiation geometry. Combining two high-Z fillers may consequently broaden the distribution of photon-interaction sites and influence secondary-photon transport [22,23,24,25]. Hybrid filler systems can also improve particle packing if differences in particle morphology or size allow one phase to occupy voids between particles of the other phase. Existing studies provide only partial evidence regarding Bi_2_O_3_/WO_3_ hybridization. Wu et al. showed that the Bi_2_O_3_ ratio influenced both gamma-ray attenuation and mechanical behavior in waterborne polyurethane composites, with attenuation varying according to photon energy and filler composition [22]. Moreover, Chai et al. developed flexible shielding materials containing tungsten and Bi_2_O_3_, demonstrating the feasibility of combining these high-Z phases [23]. Wang et al. compared elemental bismuth, tungsten, and bismuth tungstate in flexible gamma/X-ray shields and found that composition affected the energy-dependent shielding response [24]. Hybrid Bi_2_O_3_-containing epoxy systems have also been investigated with two-dimensional nanomaterials to improve filler confinement and interfacial organization [25,26]. However, many previous studies used broad diagnostic X-ray spectra or sealed gamma sources with energies that differ substantially from the 140.5 keV emission of ^99m^Tc. Others relied primarily on theoretical attenuation calculations without direct validation under a nuclear medicine exposure geometry.

Therefore, multicomponent and multilayer designs provide an additional approach to controlling photon transport. Layered polymer composites containing tungsten- and bismuth-rich phases have been reported to reduce spectral weak points associated with individual shielding elements and, under polychromatic X-ray irradiation, may facilitate reabsorption of secondary photons [27,28,29]. However, results obtained using broad X-ray spectra cannot be transferred directly to monoenergetic ^99m^Tc irradiation. Moreover, superior transmission reduction in a multilayer specimen may simply reflect increased total thickness or areal density. Rigorous comparisons must therefore control these variables and distinguish architectural effects from the additive attenuation of the component layers. Despite substantial research on single-filler Bi_2_O_3_–polymer and WO_3_–polymer composites, comparatively few studies have experimentally compared neat epoxy, Bi_2_O_3_-filled epoxy, WO_3_-filled epoxy, and hybrid Bi_2_O_3_/WO_3_-filled epoxy at the specific 140.5 keV energy of ^99m^Tc while simultaneously examining structural, morphological, interfacial, mechanical, and operational shielding performance. In particular, it remains unclear whether combining Bi_2_O_3_ and WO_3_ produces a meaningful performance advantage after accounting for total filler fraction, density, and dispersion quality. This distinction is essential for translating high-Z nanocomposites from theoretical material formulations into reproducible occupational shielding components.

Accordingly, this study synthesized Bi_2_O_3_ and WO_3_ nanoparticles and incorporated them individually into monolithic composites and sequentially into hybrid multilayer composites using a diglycidyl ether of bisphenol A (DGEBA)-based epoxy matrix. The synthesized nanoparticles, neat epoxy, and fabricated composites were characterized using complementary techniques to determine their crystalline phase composition, hydrodynamic particle-size distributions, zeta potentials, morphology, elemental composition and spatial distribution, chemical interactions, and surface wettability. The tensile response and tensile toughness of selected formulations were also evaluated to quantify maximum tensile stress, deformation capacity, and energy absorption and to compare the hybrid multilayer architecture with the reference and corresponding monolithic formulations. Their shielding performance against the 140.5 keV photons emitted by ^99m^Tc was evaluated experimentally using a fixed and reproducible source–specimen–dosimeter geometry. Background, unshielded-reference, and shielded personal dose equivalent (Hp(10)) measurements were obtained to determine radiation-shielding efficiency and the associated experimentally derived attenuation parameters. The study examined whether (i) incorporating either high-Z oxide improved photon attenuation relative to neat epoxy, (ii) the hybrid Bi_2_O_3_/WO_3_ formulation provided an additional shielding advantage over the corresponding single-filler composites, and (iii) differences in measured shielding performance could be interpreted in relation to nanoparticle dispersion, morphology, elemental distribution, and interfacial characteristics. By integrating controlled nanoparticle synthesis, comprehensive physicochemical characterization, and application-relevant ^99m^Tc dosimetry, this study provides an experimental basis for developing lead-free epoxy nanocomposites for occupational radiation protection in nuclear medicine applications.

## 2. Materials and Methods

### 2.1. Materials

Bismuth nitrate pentahydrate [Bi(NO_3_)_3_·5H_2_O; 98.0%] was obtained from KemAus (Cherrybrook, NSW, Australia). Sodium hydroxide (NaOH), sodium sulfate (Na_2_SO_4_), and tungsten oxide (WO_3_) were purchased from Thermo Fisher Scientific (Waltham, MA, USA). Bismuth oxide (Bi_2_O_3_; 99.5%, analytical reagent grade) and oxalic acid (H_2_C_2_O_4_; 99.5%, analytical reagent grade) were supplied by QReC™ (QReC Chemical Co., Ltd., Auckland, New Zealand). Sodium tungstate dihydrate (Na_2_WO_4_·2H_2_O) was obtained from Carlo Erba Reagents (Cornaredo, Milan, Italy), whereas hydrochloric acid (HCl; 37%) was purchased from RCI Labscan Limited (Pathum Wan, Bangkok, Thailand). Deionized water used throughout the experiments was produced using a Direct-Q 3 water purification system. All solvents were obtained from MilliporeSigma (St. Louis, MO, USA) or Thermo Fisher Scientific (Waltham, MA, USA).

### 2.2. Preparation, Ambient Curing, and Geometric Characterization of Monolithic and Hybrid Multilayer Epoxy Composites

#### 2.2.1. Preparation and Ambient Curing of Monolithic and Hybrid Multilayer Epoxy Composites

Bismuth(III) oxide nanoparticles (Bi_2_O_3_ NPs) were synthesized using a precipitation method. Briefly, Bi(NO_3_)_3_·5H_2_O and Na_2_SO_4_ were dissolved in 500 mL of deionized water, and the resulting solution was magnetically stirred for 2 h. Separately, NaOH was dissolved in 500 mL of deionized water to prepare an alkaline precipitating solution. The NaOH solution was added dropwise to the bismuth-containing solution at a controlled and constant rate under continuous stirring until precipitation was complete. The resulting precipitate was collected by filtration, washed with 70% ethanol, and oven-dried at 150 °C for 2 h to obtain Bi_2_O_3_ nanopowder.

Tungsten(VI) oxide nanoparticles (WO_3_ NPs) were synthesized using an acid-precipitation method. A 3.0 M HCl solution was prepared by diluting concentrated HCl (37%) with deionized water. Separately, Na_2_WO_4_·2H_2_O was dissolved in 500 mL of deionized water under continuous stirring until a clear solution was obtained. Subsequently, 100 mL of 3.0 M HCl was added dropwise to the sodium tungstate solution under continuous stirring until a yellow precipitate formed. Oxalic acid was then added, and the mixture was stirred for 1 h. Oxalic acid served as a complexing and morphology-regulating agent by coordinating tungsten-containing species and regulating nucleation and particle growth during acid precipitation. The reaction vessel was covered with aluminum foil, and the mixture was maintained at 95 °C for 12 h. The resulting precipitate was collected by filtration and oven-dried at 60 °C for 4 h to obtain WO_3_ nanopowder.

An industrial-grade E-51 bisphenol-A epoxy resin composed predominantly of diglycidyl ether of bisphenol A (DGEBA) was purchased from Sinopec Hunan Petrochemical Co., Ltd. (Yueyang, China) and used as the polymer matrix. The resin had a nominal epoxy value of 0.51 equivalents per 100 g, corresponding to an epoxy equivalent weight of 196.08 g·eq^−1^:

*EEW* = 100/0.51 = 196.08 g·eq^−1^ (1)

In addition, based on the nominal DGEBA functionality of two terminal epoxy groups per molecule, the functionality-based average molar mass was estimated as approximately 392 g·mol^−1^. The principal non-chain-extended DGEBA homologue (*n* = 0) has the molecular formula C_21_H_24_O_4_ and a molar mass of 340.42 g·mol^−1^. The generalized structure of the resin can be represented as glycidyl–O–BPA–O–[CH_2_–CH(OH)–CH_2_–O–BPA–O]_n_–glycidyl, where BPA denotes the C_6_H_4_–C(CH_3_)_2_–C_6_H_4_ bisphenol-A unit and n represents the number of chain-extension units. Furthermore, SD 8822, a commercial amine-based curing agent supplied by Sicomin Composites (Châteauneuf-les-Martigues, France), was used for ambient curing. According to the supplier’s compositional information, SD 8822 contains isophorone diamine and propoxylated polyamines that provide reactive primary and secondary amine-hydrogen functionalities. During curing, nucleophilic attack by the amine nitrogen opens the terminal oxirane rings of DGEBA, forming C–N bonds and β-hydroxyamine linkages. Repetition of these reactions progressively generates the three-dimensional crosslinked epoxy network.

Neat epoxy, monolithic nanoparticle-filled composites, and hybrid multilayer Bi_2_O_3_/WO_3_ nanoparticle-filled epoxy composites were fabricated using a conventional mixing and controlled ambient-curing procedure. The epoxy resin-to-curing-agent ratio was maintained at 3:1 by mass for all formulations, corresponding to 100 parts epoxy resin and 33.3 parts curing agent by mass. Filler loading was expressed in parts per hundred parts of epoxy resin (phr), defined as the mass of oxide nanoparticles added per 100 parts by mass of epoxy resin before addition of the curing agent. The formulations and multilayer configurations are summarized in Table 1.

For the monolithic composites, Bi_2_O_3_ NPs or WO_3_ NPs were incorporated into the epoxy resin at loadings of 25 or 50 phr. Each nanoparticle filler was added gradually to the epoxy resin and mixed continuously for 10 min to obtain a visually uniform mixture. The SD 8822 curing agent was subsequently added, and mixing was continued for an additional 5 min. Neat epoxy specimens were prepared using the same procedure without nanoparticle addition. The resulting formulations were poured into molds and cured at 25 ± 2 °C for 24 h.

Ambient curing was selected based on the chemical reactivity of the SD 8822 amine system toward the oxirane groups of the DGEBA resin. During curing, primary and secondary amine-hydrogen functionalities undergo nucleophilic addition to the epoxide groups, producing hydroxyl-containing linkages and progressively developing a three-dimensional crosslinked network [30,31]. Maintaining the curing temperature at 25 ± 2 °C, together with an identical resin-to-curing-agent ratio and curing schedule, standardized the polymerization conditions across all formulations and supported direct comparison of their physicochemical characteristics and experimentally measured radiation-shielding performance [31,32,33,34]. The fabrication protocol employed ambient curing throughout, including the sequential formation and final consolidation of the multilayer specimens.

For the hybrid multilayer composites, separate Bi_2_O_3_ NP–epoxy and WO_3_ NP–epoxy mixtures were prepared independently at either 25 or 50 phr using the same mixing procedure. The two nanoparticle systems were not premixed. The corresponding filled-epoxy formulations were sequentially cast into the molds to produce four alternating layers. The 25-phr multilayer configurations were B25/W25/B25/W25 and W25/B25/W25/B25, whereas the 50-phr configurations were B50/W50/B50/W50 and W50/B50/W50/B50. Here, B and W represent Bi_2_O_3_- and WO_3_-filled epoxy layers, respectively, and the numerical designation indicates the nanoparticle loading in each layer. Each complete multilayer assembly had a total thickness of 2.0 cm (20.0 mm). Because the four layers were deposited with approximately equal nominal thicknesses, each individual layer had a nominal thickness of approximately 0.50 cm (5.0 mm).

To maintain distinct interfaces between adjacent layers, each deposited layer was cured at 25 ± 2 °C for 24 h before application of the subsequent layer. This 24 h period was operationally defined as the interlayer stabilization interval. During this interval, crosslink-network development increased the structural stability of the deposited layer, limited macroscopic intermixing with the subsequently applied formulation, and preserved the intended Bi_2_O_3_/WO_3_ stacking sequence. Sequential deposition under identical curing conditions also promoted reproducible layer formation and maintained a consistent processing history among the four multilayer configurations.

**Figure 1:**
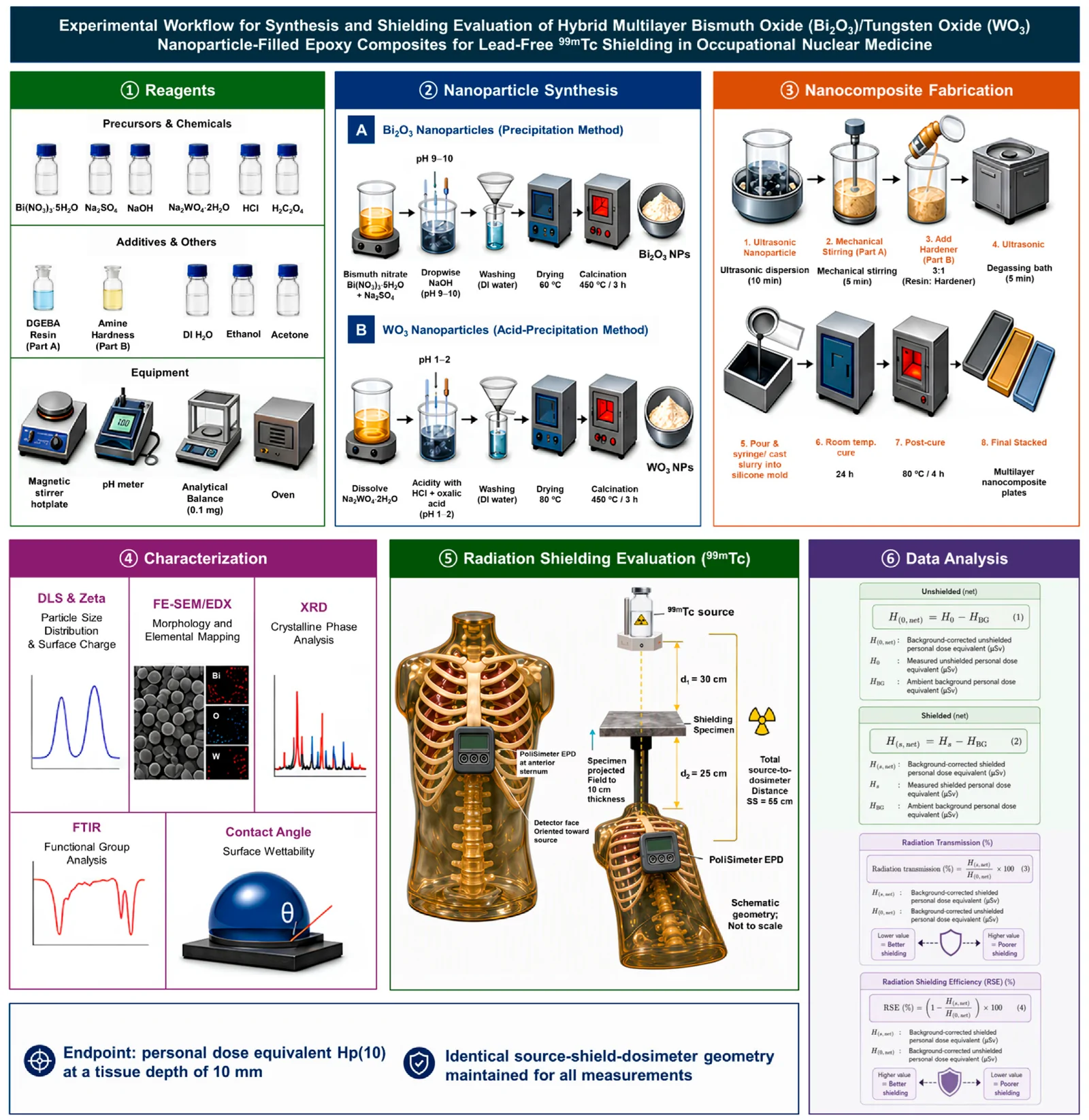
Experimental workflow for Bi_2_O_3_ and WO_3_ nanoparticle synthesis, hybrid multilayer epoxy-composite fabrication, physicochemical characterization, and ^99m^Tc shielding evaluation. Shielding measurements were performed using an anthropomorphic thorax phantom and a PoliSimeter dosimeter under a fixed source–shield–detector geometry (d_1_ = 30 cm; d_2_ = 25 cm). Shielding performance was quantified using background-corrected personal dose-equivalent readings, H_p_(10), radiation transmission, and radiation-shielding efficiency. ChatGPT (OpenAI, GPT-5.6; accessed 20 August 2026) was used to assist with the graphical rendering and layout refinement of this schematic. The authors developed and verified all scientific content, experimental parameters, labels, and final graphical elements.

After deposition of the fourth layer, the complete multilayer assembly was cured at 25 ± 2 °C for an additional 24 h to complete the fabrication cycle. The masses reported in Table 1 represent the complete specimens. Each epoxy-based specimen measured 10.0 cm in length, 10.0 cm in width, and 2.0 cm in total thickness. The specimens were subsequently removed from the molds and labelled to preserve their intended irradiation orientation. In each sequence designation, the first layer represents the surface positioned toward the ^99m^Tc source, whereas the fourth layer represents the detector-facing surface during the radiation-shielding measurements (Figure 1).

#### 2.2.2. Specimen Geometry, Geometric Bulk Density, and Areal Density

The dimensions and masses of the specimens used in the ^99m^Tc shielding measurements were recorded after completion of the ambient-curing process. The exposed frontal area, *A*, and external volume, *V*, of each epoxy-based specimen were calculated as follows:

*A = L × W* = 10.0 cm × 10.0 cm = 100.0 cm^2^

*V = L × W × t* = 10.0 cm × 10.0 cm × 2.0 cm = 200.0 cm^3^

The geometric bulk density, $\rho_{g}$, was calculated from the mass of the complete specimen and its external volume:

(2) $$\rho_{g}=\;\frac{m}{V}$$

Here, *m* is the measured specimen mass in *g*, and *V* is the external specimen volume in cm^3^. The areal density, σ, perpendicular to the incident photon beam was calculated as follows:

(3) $$\sigma =\;\frac{m}{A}$$

The areal-density calculation was independently verified using the following:

(4) $$\sigma =\;\rho_{g}\times t$$

The 0.5-mm lead reference measured 10.0 cm × 10.0 cm and had an exposed area of 100.0 cm^2^. Its nominal thickness of 0.5 mm was converted to 0.05 cm, giving an external volume of 5.00 cm^3^. The corresponding geometric and areal parameters were calculated using the same equations. Geometric bulk density describes the mass per unit external specimen volume, whereas areal density represents the total specimen mass encountered per unit area of the incident photon beam.

### 2.3. Hydrodynamic Particle-Size Distribution and Zeta Potential of Bi_2_O_3_ and WO_3_ Nanoparticle Dispersions

The hydrodynamic particle-size distributions and zeta potentials of the Bi_2_O_3_ and WO_3_ nanoparticles were determined using dynamic light scattering (DLS) and electrophoretic light scattering, respectively. Each nanoparticle sample was dispersed in deionized water at a concentration of 0.5 mg/mL. The resulting suspensions were homogenized in a bath sonicator (CPX Series Ultrasonic Cleaning Bath, Model CPX1800; Branson Ultrasonics Corporation, Brookfield, CT, USA) operating at 40 kHz with a maximum ultrasonic power of 70 W for 15 min at room temperature. During sonication, the sample tubes were fully immersed in the sonication bath to facilitate uniform ultrasonic-energy transfer and homogeneous nanoparticle dispersion.

Following sonication, each suspension was passed through a 0.2 µm polyvinylidene fluoride (PVDF) membrane filter to remove large aggregates and particulate contaminants. A 1 mL aliquot of each prepared suspension was subsequently transferred to an appropriate disposable cuvette for particle-size measurement or a disposable zeta-potential cell for electrophoretic analysis. All measurements were performed at 25 °C using a ZetaPALS analyzer (Brookhaven Instruments Corporation, Nashua, NH, USA). Particle-size measurements were conducted at a scattering angle of 173°. The refractive index and dynamic viscosity of the aqueous dispersant were set to 1.330 and 0.890 cP, respectively.

### 2.4. Tensile Mechanical Characterization and Tensile Toughness Determination

The tensile mechanical properties of the lead reference, NE, B50, W50, and ML-BW50 were evaluated using dumbbell-shaped specimens tested under uniaxial loading. Maximum force, maximum tensile stress, strain at maximum stress, terminal strain, and tensile toughness were derived from the engineering stress–strain curves. The complete experimental procedure, calculation method, curves, and numerical results are provided in Supplementary Sections S2 and S3, Figure S2, and Table S1.

### 2.5. Morphological and Elemental Characterization Using FE-SEM–EDX

The surface morphology and elemental composition of hybrid multilayer ML-BW50 samples were characterized using field-emission scanning electron microscopy coupled with energy-dispersive X-ray spectroscopy (FE-SEM–EDX). SEM micrographs were acquired using an Apreo FE-SEM system (FEI Technologies Inc., Hillsboro, OR, USA), whereas elemental analysis was performed using an EDX detector supplied by Oxford Instruments NanoAnalysis (High Wycombe, UK).

Before analysis, the samples were carefully cleaned to remove surface contaminants. Each sample was rinsed with ethanol and sonicated for 10–15 min to dislodge loosely attached contaminants and improve surface cleanliness. The samples were subsequently rinsed with deionized water and dried in a vacuum desiccator to remove residual moisture and minimize outgassing under the high-vacuum conditions used for SEM analysis. The dried samples were mounted on SEM stubs and sputter-coated with an approximately 20 nm layer of gold to enhance electrical conductivity and minimize beam-induced charging. After coating, the samples were stored in a vacuum desiccator for 15 min before analysis. Consequently, the prepared samples were introduced into the SEM chamber, and high-resolution micrographs were acquired to examine their surface morphology, particle characteristics, aggregation, and structural features. EDX analysis was subsequently performed to determine the elemental composition of each sample. Particular attention was given to detecting bismuth and oxygen in the Bi_2_O_3_-containing samples, tungsten and oxygen in the WO_3_-containing samples, and the principal elemental constituents of the neat epoxy. The EDX spectra were used to confirm the presence of the expected elements, while the corresponding elemental maps were used to evaluate their spatial distribution.

### 2.6. Structural and Crystalline-Phase Characterization by X-Ray Diffraction

The structural characteristics and crystalline phases of 0.5-mm lead reference, neat epoxy, and hybrid multilayer Bi_2_O_3_/WO_3_ NPs (ML-BW50) were investigated using XRD. Before analysis, each material was finely ground using an agate mortar and pestle to obtain a homogeneous powder with a nominal particle size of <10 µm. This preparation improved sample uniformity and minimized preferred-orientation effects during diffraction analysis. The powders were subsequently compacted into flat, uniform pellets using a hydraulic press under an applied load of 5 tons for 2 min. Each pellet was prepared with a smooth, level surface to ensure consistent diffraction measurements. Moreover, the prepared pellets were mounted in the XRD sample holder, and diffraction patterns were recorded using Cu Kα radiation (λ = 1.5406 Å) over a 2θ range of 10–90°. The crystalline phases were identified by comparing the positions and relative intensities of the observed diffraction peaks with reference patterns available in standard crystallographic databases.

### 2.7. Chemical-Bonding and Molecular-Structure Analysis by Fourier-Transform Infrared (FTIR) Spectroscopy

FTIR spectroscopy was used to investigate the molecular structures and chemical-bonding characteristics of the prepared materials. Spectra were acquired using a VERTEX 70 FTIR spectrometer (Bruker Daltonics GmbH & Co. KG, Bremen, Germany) equipped with an attenuated total reflectance (ATR) accessory and a deuterated L-alanine-doped triglycine sulfate (DLaTGS) detector with potassium bromide (KBr) windows. Each spectrum was recorded over the wavenumber range of 4000–400 cm^−1^ by co-adding 64 scans. Additionally, the spectra were examined to identify the characteristic absorption bands associated with the functional groups and chemical bonds present in the neat epoxy, nanoparticle fillers, and composite samples. Particular attention was given to changes in band position, intensity, and shape, as well as the appearance or disappearance of absorption bands, because these changes may indicate chemical interactions, structural alterations, or modifications to the bonding environment within the composite materials.

### 2.8. Surface Wettability Assessed by Static Phosphate-Buffered Saline Contact-Angle Measurements

The surface wettability of NE, B50, W50, ML-BW50, and the 0.5-mm lead reference was evaluated using an OCA 25 optical contact-angle analyzer (DataPhysics Instruments GmbH, Filderstadt, Germany). Phosphate-buffered saline (PBS) was used as a nonradioactive aqueous simulant of the vehicle used for the ^99m^Tc solution. Static PBS contact angles were measured using the sessile-drop method under controlled laboratory conditions of 23 ± 2 °C and 50 ± 10% relative humidity.

Before measurement, the specimens were handled with clean forceps to avoid contact with the measurement areas. Loose surface particles were removed using a gentle stream of dry, filtered nitrogen, and each specimen was positioned horizontally on the instrument stage. Calcium- and magnesium-free 1× PBS containing 137 mM NaCl, 2.7 mM KCl, 10 mM Na_2_HPO_4_, and 1.8 mM KH_2_PO_4_ was freshly prepared using deionized water and adjusted to pH 7.4 ± 0.1. The PBS was equilibrated to 23 ± 2 °C before being loaded into the dispensing syringe. A 5 µL droplet was formed at the needle tip, after which the sample stage was raised slowly until the specimen contacted the suspended droplet. The stage was then lowered carefully to transfer the droplet without allowing the needle to contact the specimen surface. Droplet images were acquired 5 s after deposition and analyzed using dpiMAX software version 2.0.118 (DataPhysics Instruments GmbH, Filderstadt, Germany). The software automatically identified the solid–liquid baseline and droplet contour, and the contact angles were calculated using an elliptical drop-profile fitting model. The left and right contact angles were recorded separately to assess droplet symmetry, and their arithmetic mean was calculated as the overall contact angle for each droplet. Three fresh PBS droplets were analyzed at separate, non-overlapping locations on each material, and previously wetted areas were not reused. Results were expressed as the mean ± SD of three droplet measurements (*n* = 3).

### 2.9. Radiation-Shielding Performance and Hp(10) Measurements

**Figure 2:**
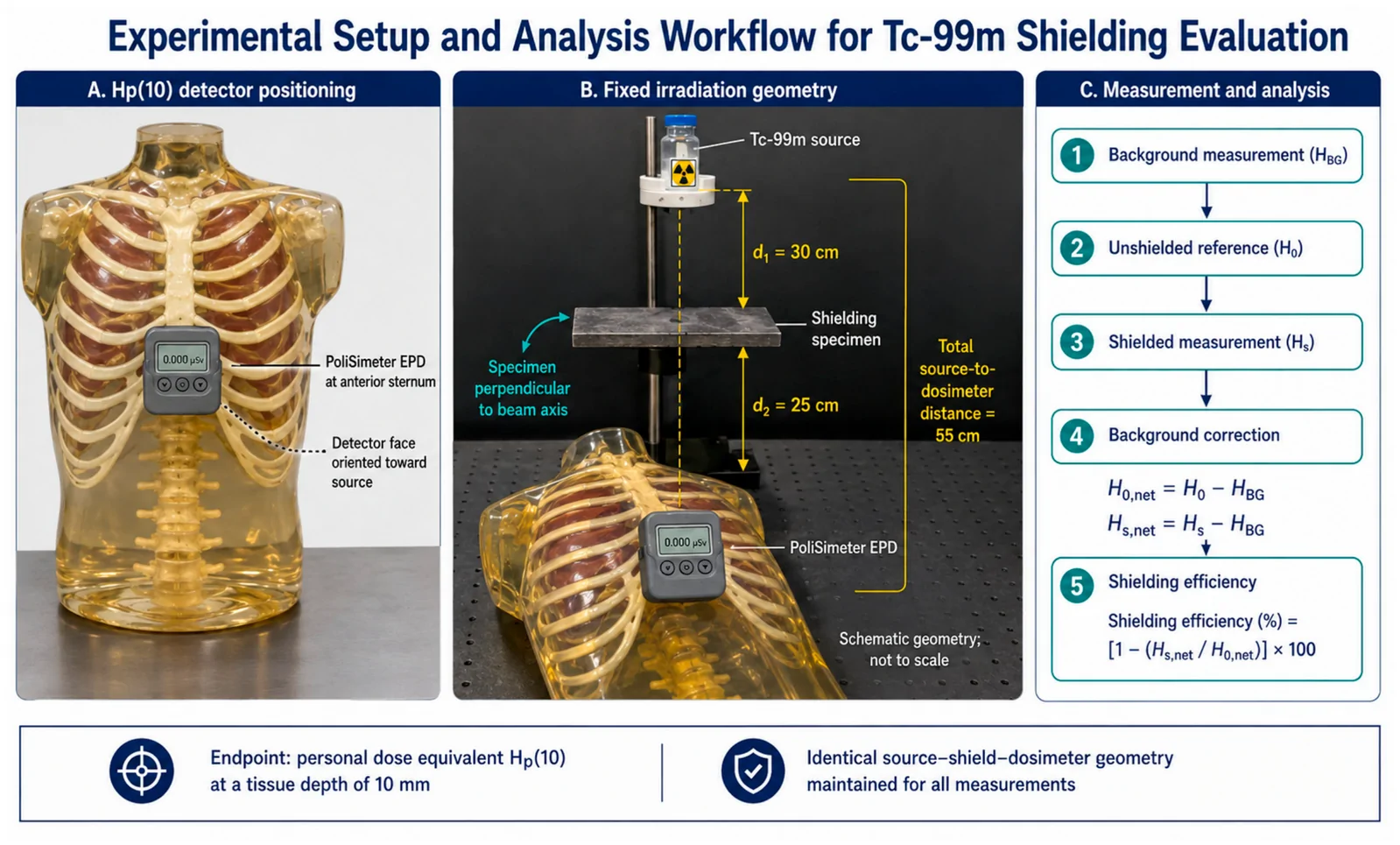
Experimental arrangement and analytical workflow used to evaluate shielding performance at ^99m^Tc activity levels of 925 MBq (25 mCi) and 6.105 GBq (165 mCi). The PoliSimeter electronic personal dosimeter was positioned over the anterior sternum of the transparent RS-111T anthropomorphic thorax phantom, with its detector face oriented toward the radiation source. The source, shielding specimen, and dosimeter were aligned along a common central axis, with the shielding specimen positioned perpendicular to this axis. The source-to-shield distance (d_1_) and shield-to-dosimeter distance (d_2_) were maintained at 30 and 25 cm, respectively, providing a total source-to-dosimeter distance of 55 cm. Background, unshielded-reference, and shielded measurements were obtained using identical irradiation geometry. Background-corrected measurements were used to calculate radiation transmission and shielding efficiency separately for each activity level. The measurement endpoint was the personal dose equivalent (Hp(10)), corresponding to a tissue depth of 10 mm. The illustrated geometry is not drawn to scale.

Radiation-shielding performance was evaluated at two nominal ^99m^Tc activity levels: 925 MBq (25 mCi) and 6.105 GBq (165 mCi). The experimental system consisted of a vial containing the ^99m^Tc radiopharmaceutical, the prepared shielding specimen, a PoliSimeter™ electronic personal dosimeter (Polimaster Europe UAB, Vilnius District, Lithuania), and a transparent anthropomorphic thorax phantom (model RS-111T; Radiology Support Devices, Inc., Gardena, CA, USA). The PoliSimeter was configured to measure the accumulated personal dose equivalent, Hp(10), expressed in µSv. Hp(10) is an operational radiation-protection quantity representing the dose equivalent in ICRU soft tissue at a depth of 10 mm below a specified point on the body and is used for individual monitoring of strongly penetrating photon radiation. The thorax phantom was positioned supine on the experimental platform. The PoliSimeter was securely attached to the anterior midline of the sternum, with its detector face oriented toward the radiation source. The ^99m^Tc source, shielding specimen, and dosimeter were aligned along a common central axis. Each shielding specimen was centered between the source and dosimeter and positioned perpendicular to the source–detector axis. The source-to-shield distance (d_1_) was fixed at 30 cm, whereas the shield-to-dosimeter distance (d_2_) was maintained at 25 cm, resulting in a total source-to-dosimeter distance of 55 cm. The specimen surface covering the central radiation field was maintained in the same position for all measurements, and the irradiation orientation of each multilayer specimen was preserved according to its designated source-to-detector layer sequence (Figure 2).

Before each activity-specific measurement series, the operational status of the dosimeter was verified, and the accumulated-dose display was reset to zero. An ambient background measurement ($H_{BG}$) was recorded for 30 s, corresponding to the exposure duration used for the experimental measurements. For each ^99m^Tc activity condition, an unshielded reference measurement ($H_{0}$) was obtained without a shielding specimen in the radiation path. Shielded measurements ($H_{S}$) were subsequently performed with each specimen positioned between the source and dosimeter. Each measurement generated an independently recorded accumulated Hp(10) value over an exposure period of 30 s. Five replicate measurements were obtained for every experimental condition (*n* = 5). The source position, specimen orientation, phantom position, detector orientation, exposure duration, and source–shield–dosimeter distances were maintained throughout the corresponding unshielded and shielded measurements. The ^99m^Tc activity at the beginning of every exposure was recorded to account for radioactive decay during sequential data acquisition.

The measured Hp(10) values were corrected for ambient background as follows:

(5) $$H_{0,C}\;=\;H_{0}-H_{BG}$$

(6) $$H_{S,C}=H_{S}-H_{BG}$$

where $H_{0,C}\;$ and $H_{S,C}$ are the background-corrected unshielded and shielded personal dose equivalents, respectively; $H_{0}\;$ is the measured unshielded Hp(10); $H_{S}$ is the measured shielded Hp(10); and $H_{BG}$ is the ambient-background Hp(10) recorded over the same 30-s interval.

To account for the physical decay of ^99m^Tc during sequential measurements, the activity at the beginning of each exposure was related to the activity at the designated reference time according to the following:

(7) $$A_{t}=\;A_{ref}e^{-\lambda t}$$

(8) $$\lambda =\frac{ln(2)}{T_{1/2}}$$

Here, $A_{t}$ is the activity at the beginning of the exposure, $A_{ref}$ is the activity at the designated reference time, Δt is the elapsed time from the reference time, λ is the physical decay constant, and T_1_/_2_ is the physical half-life of ^99m^Tc (6 h). The background-corrected Hp(10) values were normalized to the corresponding reference activity using the following:

(9) $$H_{norm}=\;H_{C}\times \left[\frac{A_{ref}}{A_{t}}\right]$$

Here, $H_{C}$ represents either $H_{0,C}$ or $H_{S,C}$, and $H_{norm}$ is the corresponding decay-normalized personal dose equivalent. This normalization placed measurements acquired at different times within the same activity series on a common activity basis.

Experimental radiation transmission and radiation-shielding efficiency (RSE) were calculated independently for each activity condition using the decay-normalized shielded and unshielded values:

(10) $$T_{exp}=\frac{H_{S,norm}}{H_{0,norm}}$$

(11) $$SE\left(\%\right)=\left[1-T_{exp}\right]\times 100$$

Here, $T_{exp}$ is the experimentally determined transmission fraction, $H_{S,norm}$ is the background-corrected and decay-normalized Hp(10) measured with the shielding specimen, and $H_{0,norm}$ is the corresponding background-corrected and decay-normalized unshielded reference. Radiation transmission is dimensionless, whereas RSE is expressed as a percentage.

Each RSE value was therefore normalized to the background-corrected unshielded reference obtained for the corresponding ^99m^Tc activity series. This activity-specific calculation preserves direct comparison among the shielding materials under each exposure condition and incorporates the measured dosimetric response of the complete source–shield–phantom–dosimeter arrangement. Results were expressed as the mean ± standard deviation of five replicate measurements (*n* = 5).

### 2.10. Statistical Analysis

Statistical analyses were performed using GraphPad Prism version 10.0 (GraphPad Software, LLC, Boston, MA, USA). Data are presented as the mean ± SD from three to ten independent replicates, depending on the experimental endpoint; the exact sample size (*n*) is reported in the corresponding figure legend. For the radiation-shielding analysis, the background-corrected personal dose equivalent, Hp(10), was the primary outcome. Data obtained at 925 MBq (25 mCi) and 6.105 GBq (165 mCi) were analyzed separately. At each activity level, differences among shielding formulations were evaluated using ordinary one-way analysis of variance (ANOVA), followed by Tukey’s multiple-comparisons test. Welch’s one-way ANOVA, followed by the Games–Howell multiple-comparisons test, was additionally performed as an unequal-variance sensitivity analysis. Radiation transmission and radiation-shielding efficiency (RSE) were calculated from the background-corrected dose equivalent and reported descriptively. Because these two parameters are mathematically complementary, they were not treated as independent statistical outcomes. All tests were two-sided, and statistical significance was defined as *p* < 0.05. Statistical annotations used in the figures were defined as follows: ns, *p* ≥ 0.05; *, *p* < 0.05. Values below the reporting threshold were presented as *p* < 0.0001.

## 3. Results

### 3.1. Integrated Physicochemical and Microstructural Analysis of Bi_2_O_3_/WO_3_ Nanoparticle-Filled Epoxy Composites

#### 3.1.1. Hydrodynamic Diameter, Cumulant Polydispersity Index, and Zeta Potential of Bi_2_O_3_ and WO_3_ Dispersions

**Figure 3:**
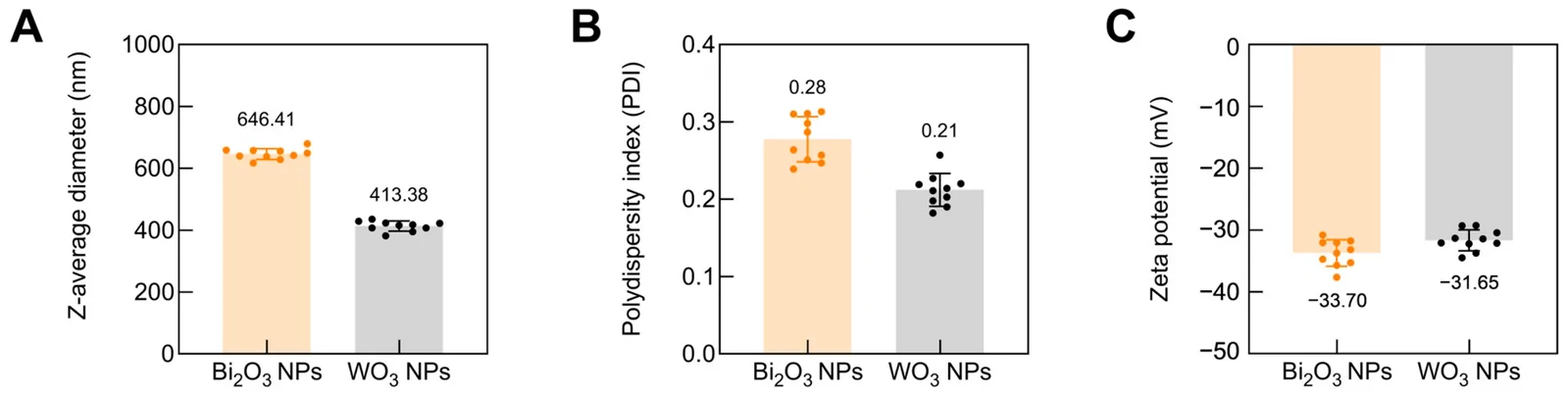
Colloidal dispersion-state characterization of the synthesized Bi_2_O_3_ and WO_3_ particles. (**A**) Cumulant-derived Z-average hydrodynamic diameter determined by dynamic light scattering. (**B**) Cumulant polydispersity index (PDI). (**C**) Zeta potential determined from electrophoretic mobility. Bars represent mean values, error bars represent standard deviations, and superimposed points represent individual replicate measurements (*n* = 10 per oxide dispersion). The Z-average values describe the equivalent-sphere hydrodynamic diameters of the scattering entities present in the filtered dispersions.

Dynamic light scattering analysis yielded Z-average hydrodynamic diameters of 646.41 nm for the Bi_2_O_3_ dispersion and 413.38 nm for the WO_3_ dispersion (Figure 3). The WO_3_ Z-average was therefore 233.03 nm lower than that of Bi_2_O_3_, corresponding to a relative difference of 36.1% (Figure 3A). The Z-average is a cumulant-derived, intensity-weighted hydrodynamic diameter calculated from the translational-diffusion behavior of the scattering entities. It represents the diameter of an equivalent sphere with the same diffusion coefficient and incorporates the hydrodynamic contribution of the solid particle or associated particle population and its coupled liquid layer [35,36]. Consequently, the reported values characterize the dispersion state at the time of measurement and do not constitute direct measurements of individual solid primary-particle dimensions. Moreover, the Z-average diameters exceeded the nominal 200 nm pore rating of the membrane used during sample preparation. This observation reflects the different physical quantities represented by membrane-pore rating and DLS-derived hydrodynamic diameter. A nominal 0.20 µm membrane does not act as a uniform geometric cutoff composed of identically sized pores. Membranes possess distributions of effective pore dimensions, while particle retention is influenced by pore geometry, membrane structure, particle morphology and orientation, filtration pressure, suspension concentration, particle–membrane interactions, and progressive pore blocking. Experimental microfiltration studies have demonstrated that particles exceeding a membrane’s nominal pore dimension can remain in the filtrate and that filtration can modify particle concentration, cumulant diameter, and PDI [37]. Particle geometry also affects the relationship between membrane passage and hydrodynamic diameter. Passage through a membrane is governed by the particle dimension and orientation presented to the pore entrance, whereas DLS reports an equivalent-sphere diameter determined by translational friction. An irregular, anisotropic, porous, or loosely associated scattering entity may pass through a pore according to its smaller projected dimension and subsequently exhibit a larger hydrodynamic diameter in the measurement medium. Primary particles or small particle associations capable of passing through the membrane may also form larger hydrodynamic entities during the interval between filtration and DLS acquisition. Time-dependent association of metal and metal-oxide particles in liquid media has been reported previously and is influenced by the dispersion medium, particle concentration, sonication history, and elapsed time after preparation [36,38]. The optical weighting of DLS further contributes to Z-average values above the nominal membrane-pore dimension. Scattered-light intensity increases strongly with particle size and, under Rayleigh-scattering conditions, is approximately proportional to the sixth power of particle diameter: I_scattering_ α d^6^. Accordingly, doubling the particle diameter can increase its scattering contribution by approximately 64-fold within the Rayleigh approximation. At dimensions approaching the wavelength of the incident laser, Mie-scattering behavior becomes applicable; however, the strong size dependence of the detected scattering signal remains. A numerically small subpopulation of larger scattering entities can therefore make a disproportionately large contribution to the autocorrelation function and increase the cumulant-derived Z-average [35,36,39]. The measured diameters of 646.41 and 413.38 nm consequently describe the hydrodynamic association states of the particle populations present in the filtered analytical dispersions.

The cumulant polydispersity index was lower for WO_3_ than for Bi_2_O_3_ (0.21 and 0.28, respectively; Figure 3B). Under the identical sample-preparation and measurement conditions applied to both oxides, the lower WO_3_ PDI indicates a lower apparent breadth of the diffusion-decay distribution. This observation is consistent with the smaller WO_3_ Z-average measured in the same analytical series. The PDI is reported as a cumulant-derived dispersion-state descriptor and is evaluated together with the individual replicate measurements because it is not a direct microscopy-based particle-size distribution [35,36].

Electrophoretic-light-scattering measurements produced zeta potentials of −33.70 mV for Bi_2_O_3_ and −31.65 mV for WO_3_ (Figure 3C). These negative values establish negative electrokinetic potentials at the hydrodynamic slipping planes of both oxide populations under the applied measurement conditions. The difference between their mean zeta potentials was 2.05 mV, indicating comparable electrokinetic behavior in the measurement medium.

Accordingly, under identical sample-preparation and measurement conditions, the WO_3_ dispersion exhibited a Z-average hydrodynamic diameter of 413.38 nm, representing a decrease of 233.03 nm (36.1%) relative to the 646.41 nm measured for Bi_2_O_3_. The corresponding cumulant PDI was also lower for WO_3_ than for Bi_2_O_3_ (0.21 versus 0.28), consistent with a lower apparent breadth of the translational-diffusion distribution in the filtered dispersion. By comparison, the zeta potentials of Bi_2_O_3_ and WO_3_ were −33.70 and −31.65 mV, respectively, with an absolute difference of 2.05 mV, demonstrating closely comparable electrokinetic characteristics under the applied analytical conditions. Overall, the principal differences between the two oxide dispersions were associated with their hydrodynamic dimensions and apparent dispersity, whereas their measured electrokinetic potentials were similar.

#### 3.1.2. Tensile Properties and Tensile Toughness

The selected materials exhibited distinct tensile responses, as presented in Supplementary Figure S2 and Table S1. ML-BW50 exhibited a maximum tensile stress of 4.008 MPa, a terminal strain of 74.294%, and a tensile toughness of 2.250 MJ·m^−3^. Relative to B50 and W50, ML-BW50 showed increases of 7.7% and 38.3% in maximum tensile stress, 18.3% and 14.9% in terminal strain, and 16.7% and 43.2% in tensile toughness, respectively. Its tensile toughness also exceeded that of neat epoxy by 4.0%, representing the highest measured value among the investigated epoxy-based formulations. These findings demonstrate that the alternating Bi_2_O_3_/WO_3_ multilayer architecture provided a favorable combination of tensile strength, deformability, and tensile-energy absorption compared with the corresponding monolithic nanoparticle-filled composites.

#### 3.1.3. Cross-Sectional Morphology and Elemental Distribution of the ML-BW50 Composite

**Figure 4:**
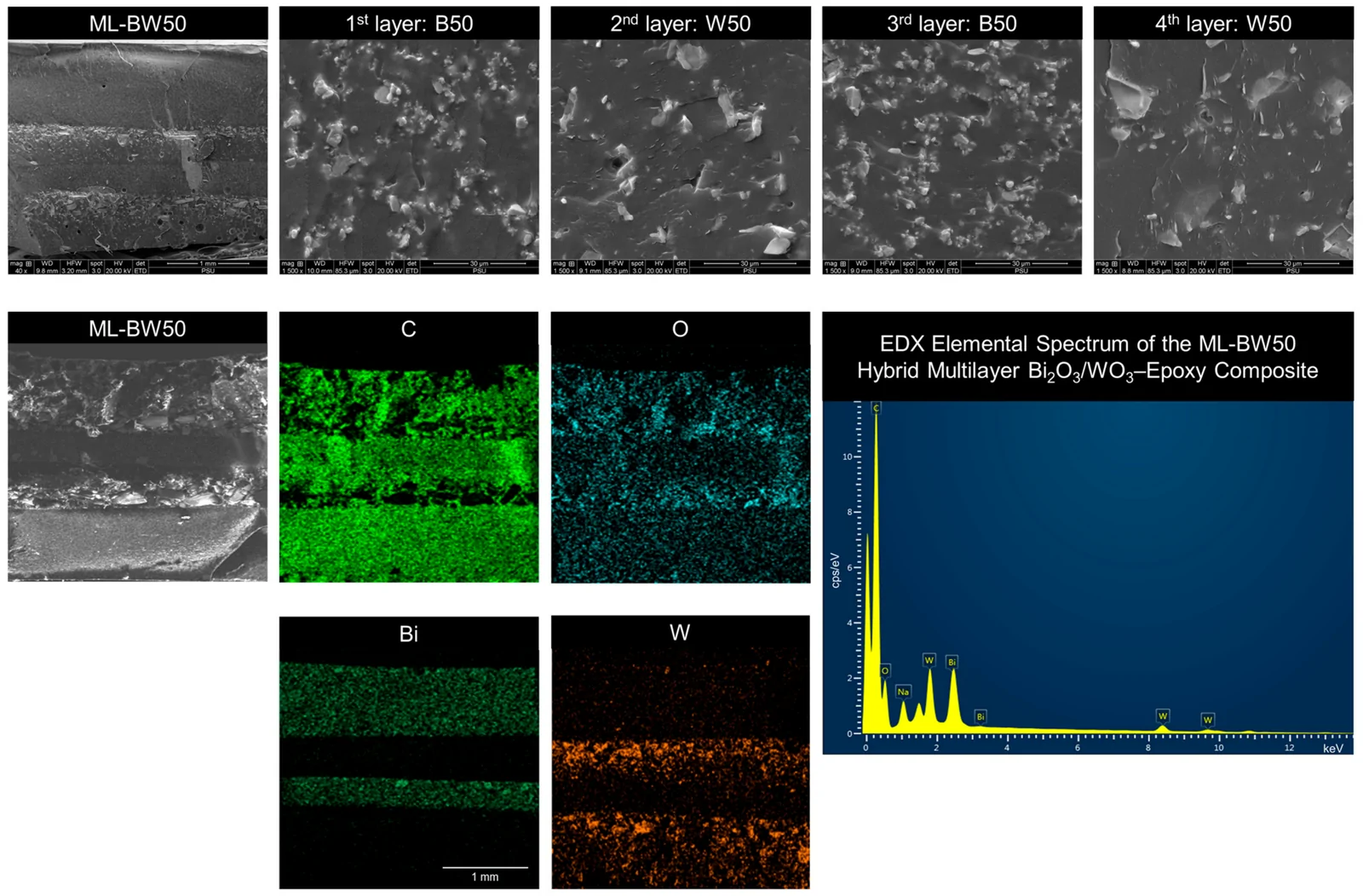
Cross-sectional FE-SEM morphology and EDX elemental analysis of the B50/W50/B50/W50 hybrid multilayer epoxy composite (ML-BW50). The upper panels show the complete multilayer cross-section at low magnification and higher-magnification micrographs of the first B50, second W50, third B50, and fourth W50 layers. B50 and W50 denote epoxy layers prepared with 50 phr Bi_2_O_3_- and WO_3_-based nanofillers, respectively. The first layer represents the source-facing surface, whereas the fourth layer represents the detector-facing surface. The lower panels show the cross-sectional region examined by EDX, the corresponding C, O, Bi, and W elemental maps, and the representative EDX spectrum. Scale bars are 1 mm for the low-magnification cross-sectional image and elemental maps and 30 µm for the individual-layer micrographs.

The cross-sectional FE-SEM micrographs in Figure 4 show that the intended B50/W50/B50/W50 architecture of ML-BW50 was retained following sequential casting and curing. At low magnification, the four constituent layers remained distinguishable, and their interfaces were generally continuous, without extensive delamination within the examined field of view. However, localized pores, apparent particle-pull-out cavities, and filler-rich agglomerates were visible, particularly near some interlayer boundaries. These features may be associated with air entrapment, increased mixture viscosity at a filler loading of 50 phr, and incomplete breakup of nanoparticle agglomerates during processing. Similar agglomeration at high Bi_2_O_3_ concentrations has been reported in Bi_2_O_3_/epoxy composites [40]. At higher magnification, the first and third B50 layers contained predominantly fine, irregular granular agglomerates embedded within the epoxy matrix. In contrast, the second and fourth W50 layers exhibited comparatively larger angular or plate-like particles, together with occasional cavities and locally heterogeneous filler-rich regions.

The EDX spectrum detected C, O, Na, Bi, and W. The dominant carbon signal was consistent with the epoxy matrix, whereas oxygen originated from both the polymer network and the oxygen-containing inorganic phases. The Bi and W peaks confirmed the incorporation of both high-atomic-number filler systems. The Na signal was compatible with the presence of a sodium-containing phase; however, its specific assignment to Na_2_WO_4_·2H_2_O is supported by the complementary XRD results because EDX alone cannot determine crystalline phase or oxidation state. Elemental mapping showed that carbon and oxygen were distributed throughout the cross-section, consistent with the continuity of the epoxy matrix and the widespread presence of oxygen-containing phases. More importantly, the Bi and W maps exhibited spatially complementary banded distributions: Bi was concentrated predominantly within the first and third layers, whereas W enrichment occurred mainly within the second and fourth layers. This pattern provides direct compositional evidence that the designed alternating B50/W50/B50/W50 sequence was preserved after curing. Limited overlap between the Bi and W signals near some boundaries may reflect localized interfacial mixing during sequential casting, together with the finite X-ray interaction volume of the EDX analysis. The observed architecture ensured that radiation traversed alternating Bi- and W-enriched regions. This provides structural context for the enhanced ^99m^Tc attenuation of ML-BW50, because particle size and dispersion influence the attenuation performance of Bi_2_O_3_/epoxy composites [18], and multilayer systems containing heavy-metal oxides have demonstrated greater attenuation than corresponding single-layer structures [29]. Altogether, the FE-SEM and EDX findings verify successful fabrication of a compositionally differentiated multilayer structure and provide microstructural support for the enhanced ^99m^Tc attenuation subsequently observed for ML-BW50.

#### 3.1.4. X-Ray Diffraction Analysis of the Crystalline Phase Composition of Hybrid Multilayer Bi_2_O_3_/WO_3_ Nanoparticle-Filled Epoxy Composites

**Figure 5:**
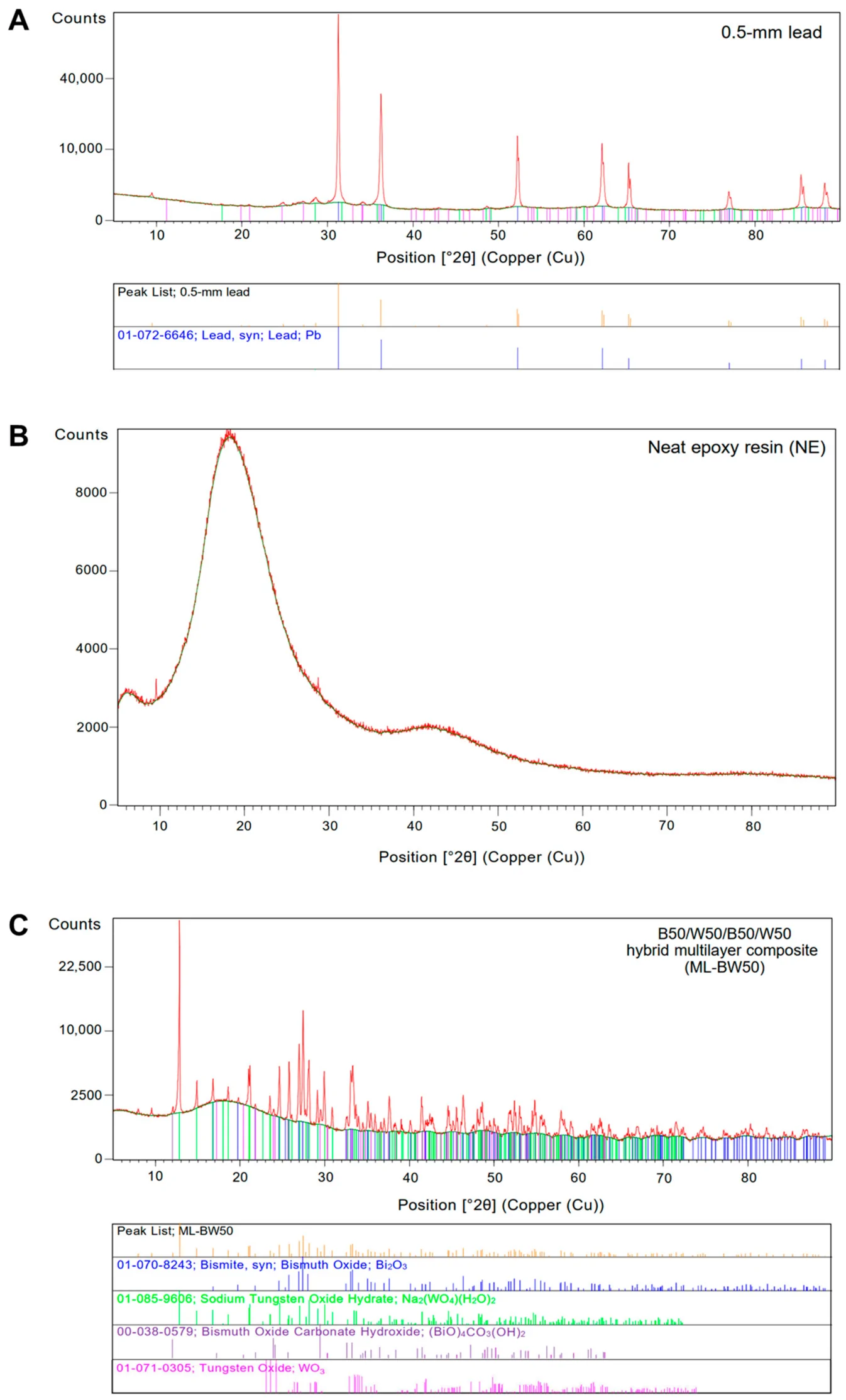
X-ray diffraction patterns of (**A**) the 0.5-mm lead reference, (**B**) neat epoxy resin (NE), and (**C**) the B50/W50/B50/W50 hybrid multilayer composite (ML-BW50), obtained using Cu Kα radiation.

Figure 5 compares the X-ray diffraction patterns of the 0.5-mm lead reference, neat epoxy resin (NE), and the ML-BW50 hybrid multilayer composite. The lead reference exhibited sharp reflections at approximately 31.3°, 36.3°, 52.3°, 62.2°, 65.3°, 77.0°, 85.5°, and 88.4° 2θ, consistent with crystalline metallic Pb (ICDD 01-072-6646). NE exhibited a broad diffuse maximum centered at approximately 18–20° 2θ and no prominent discrete Bragg reflections, consistent with the predominantly amorphous structure of the cured epoxy matrix. The ML-BW50 pattern contained the broad epoxy contribution together with numerous sharp reflections, demonstrating the presence of crystalline filler-derived phases within the cured composite.

Qualitative reference-pattern matching of ML-BW50 identified diffraction features consistent with four crystalline phases: monoclinic α-Bi_2_O_3_ (ICDD 01-070-8243), bismuth oxide carbonate hydroxide, (BiO)_4_CO_3_(OH)_2_ (ICDD 00-038-0579), tungsten trioxide, WO_3_ (ICDD 01-071-0305), and sodium tungstate dihydrate, Na_2_WO_4_·2H_2_O (ICDD 01-085-9606). The simultaneous detection of WO_3_ and Na_2_WO_4_·2H_2_O indicates partial conversion of the sodium tungstate precursor during preparation of the W-containing powder. The synthesis involved acid precipitation, aging at 95 °C for 12 h, and subsequent drying at 60 °C for 4 h. Previous studies of acidified sodium tungstate systems have reported the formation of hydrated tungsten-containing intermediates, followed by crystallization of WO_3_ during subsequent thermal treatment [41,42,43]. The concurrent identification of α-Bi_2_O_3_ and (BiO)_4_CO_3_(OH)_2_ demonstrates the presence of a mixed Bi-containing crystalline phase assemblage within ML-BW50. Bismuth oxycarbonate and hydroxycarbonate phases have previously been observed in aqueous alkaline Bi-containing systems, with phase development governed by solution chemistry and thermal treatment [44,45,46]. Collectively, the XRD data identify ML-BW50 as an amorphous epoxy matrix containing mixed-phase Bi- and W-containing crystalline constituents. Accordingly, the radiation-shielding results for ML-BW50 reflect the performance of the complete fabricated composite, encompassing its crystalline phase assemblage, epoxy matrix, and multilayer architecture.

#### 3.1.5. FTIR Characterization of Hybrid Multilayer Bi_2_O_3_/WO_3_ Nanoparticle-Filled Epoxy Composites

**Figure 6:**
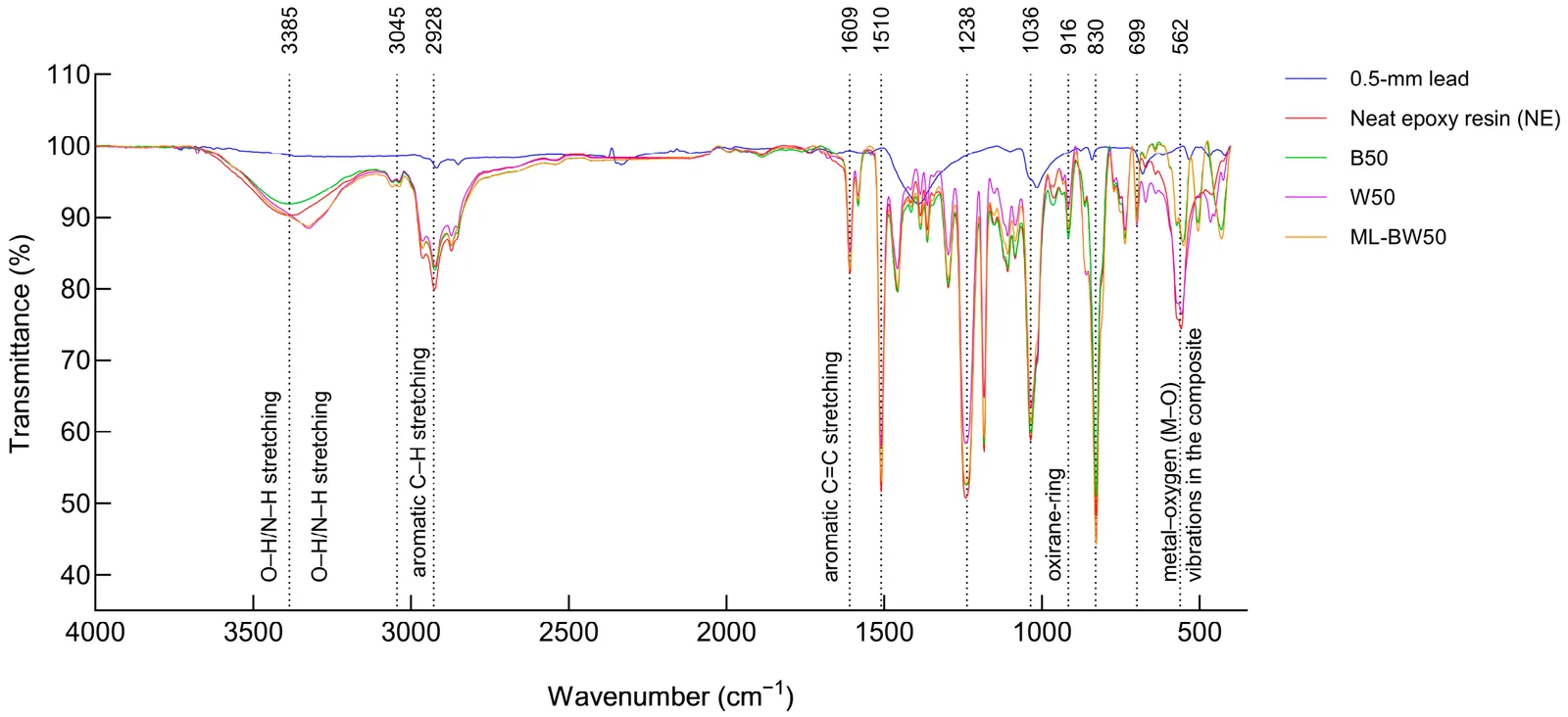
FTIR transmittance spectra of the 0.5-mm lead reference, neat epoxy resin (NE), Bi_2_O_3_-filled epoxy containing 50 phr filler (B50), WO_3_-filled epoxy containing 50 phr filler (W50), and the B50/W50/B50/W50 hybrid multilayer composite (ML-BW50), recorded over the range of 4000–400 cm^−1^. Downward spectral features represent absorption bands in the transmittance (%) presentation.

The FTIR spectra of 0.5-mm lead, neat epoxy resin (NE), B50, W50, and ML-BW50 exhibited the characteristic absorption pattern of the cured epoxy network (Figure 6). The broad band centered at approximately 3385 cm^−1^ was assigned to O–H stretching, with possible contributions from residual N–H groups. The bands at 3045 and 2928 cm^−1^ were attributed to aromatic and aliphatic C–H stretching, respectively. Aromatic-ring vibrations were observed at 1609 and 1510 cm^−1^, while the bands at 1238 and 1036 cm^−1^ were associated predominantly with ether and C–O stretching within the epoxy network. The feature near 916 cm^−1^ was assigned to an oxirane-ring vibration, and the band at approximately 830 cm^−1^ was attributed mainly to out-of-plane C–H bending of para-substituted aromatic rings. The retention of these principal bands in B50, W50, and ML-BW50 indicates that the characteristic chemical structure of the epoxy matrix was preserved following nanoparticle incorporation and multilayer fabrication. Additionally, the differences among the filler-containing spectra were most evident in the low-wavenumber region. Changes around 830–699 cm^−1^ are consistent with contributions from W–O and W–O–W vibrations, whereas the feature near 562 cm^−1^ may include Bi–O lattice vibrations. As a result, ML-BW50 retained the epoxy-related bands while also displaying low-wavenumber contributions associated with the metal-oxide fillers, supporting their incorporation within the composite structure.

#### 3.1.6. Surface Wettability of Bi_2_O_3_- and WO_3_-Nanoparticle-Filled Epoxy Resin Composites Toward Phosphate-Buffered Saline

**Figure 7:**
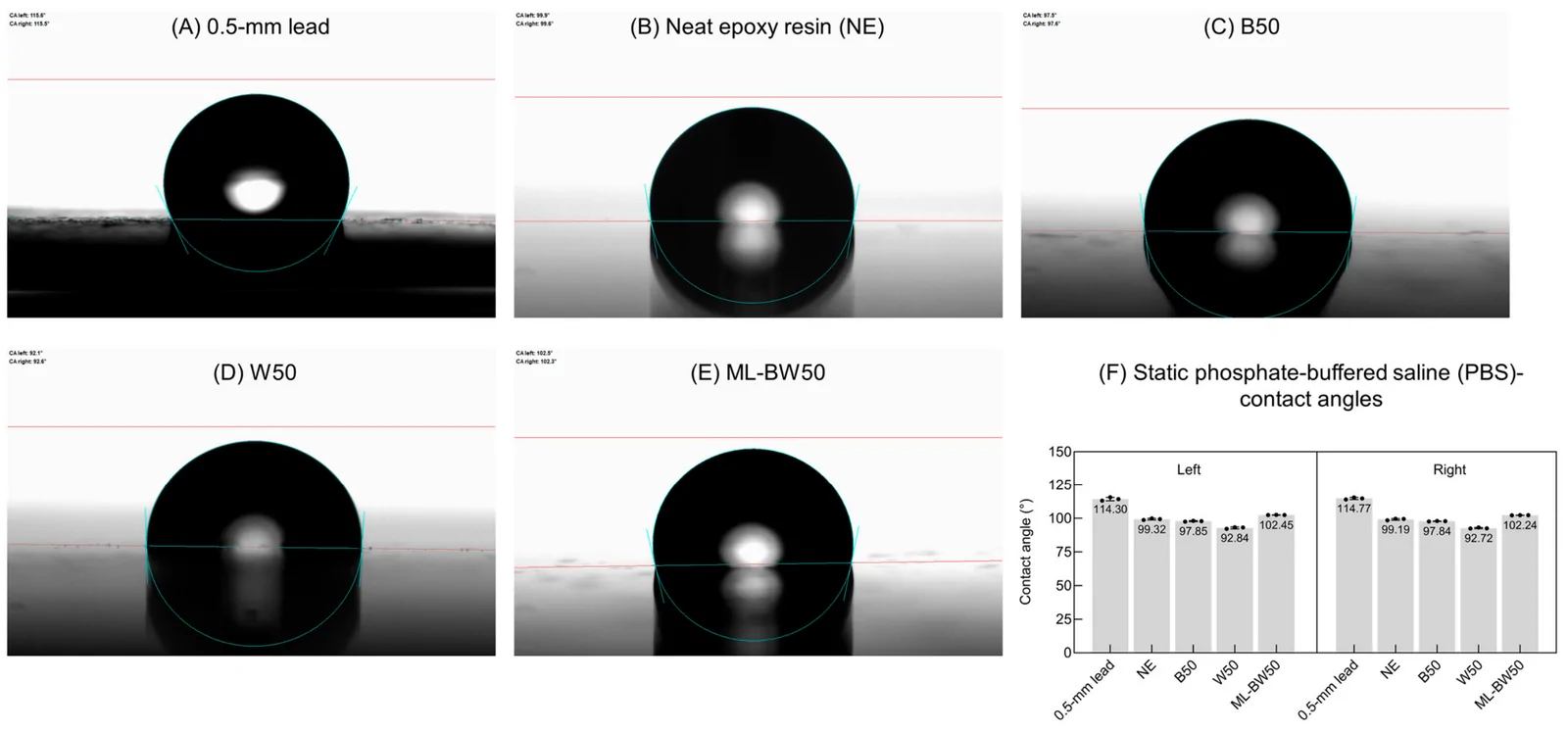
Static phosphate-buffered saline (PBS) contact angles of the 0.5-mm lead reference and Bi_2_O_3_- and WO_3_-nanoparticle-filled epoxy resin composites measured using the sessile-drop method. Nonradioactive PBS was used as an aqueous simulant of the medium used for the ^99m^Tc solution. Representative droplet profiles and fitted contact-angle boundaries are shown for (**A**) 0.5-mm lead, (**B**) neat epoxy resin (NE), (**C**) Bi_2_O_3_-filled epoxy containing 50 phr filler (B50), (**D**) WO_3_-filled epoxy containing 50 phr filler (W50), and (**E**) the B50/W50/B50/W50 hybrid multilayer composite (ML-BW50). (**F**) Quantitative comparison of the left- and right-side PBS contact angles. Bars represent the mean of three replicate measurements, black circles indicate individual measurements, and numerical labels report the corresponding mean values in degrees.

Figure 7 presents representative sessile-drop profiles and quantitative left- and right-side static contact angles measured using PBS. PBS was selected as a nonradioactive aqueous simulant of the medium used for the ^99m^Tc solution. All mean contact angles exceeded 90°, indicating limited surface wetting by PBS. The 0.5-mm lead reference exhibited the highest mean contact angles, with values of 114.30° and 114.77° on the left and right sides, respectively. The corresponding values were 99.32° and 99.19° for neat epoxy resin, 97.85° and 97.84° for B50, and 92.84° and 92.72° for W50. These results indicate that nanoparticle incorporation altered the interaction between the PBS droplets and the epoxy surfaces, with WO_3_ associated with greater PBS wettability than Bi_2_O_3_. The difference between the left- and right-side contact angles did not exceed 0.47° for any material, supporting good droplet symmetry and internal measurement consistency. Therefore, ML-BW50 exhibited mean left- and right-side PBS contact angles of 102.45° and 102.24°, respectively, making it the least wettable epoxy-based formulation examined. A relatively high PBS contact angle promotes a more compact droplet and restricts lateral spreading. In the event of an accidental liquid ^99m^Tc spill, this behavior could reduce the contaminated surface area and potentially facilitate localization, recovery, and removal by wiping. As a result, ML-BW50 provides a useful combination of gamma-ray attenuation and surface properties favorable for contamination control.

### 3.2. Radiation-Shielding Properties of Hybrid Multilayer Bi_2_O_3_/WO_3_ Nanoparticle-Filled Epoxy Resin Composites

As shown in Table 2, the tested shielding materials produced markedly different background-corrected personal dose equivalent at both 925 MBq (25 mCi) and 6.105 GBq (165 mCi). The no-shield control produced a mean personal dose equivalent of 146.77 ± 3.45 µSv, whereas NE produced 151.35 ± 3.33 µSv. This corresponded to 103.12% transmission and a calculated shielding efficiency of −3.12%. The small negative value does not indicate that NE generated or increased radiation; rather, it reflects normal measurement variation between the NE and no-shield measurements. Therefore, NE provided no measurable attenuation at this activity. In contrast, 0.5-mm lead reduced the mean dose to 35.56 µSv, corresponding to 24.23% transmission and 75.77% shielding efficiency. ML-BW50 produced the lowest mean dose of 33.15 µSv, with 22.58% transmission and 77.42% shielding efficiency. Thus, ML-BW50 provided a numerical shielding-efficiency advantage of approximately 1.65 percentage points over lead at 925 MBq. A similar performance pattern was observed at 6.105 GBq. The no-shield control produced a mean dose of 1320.60 ± 10.32 µSv, while NE reduced it to 1186.45 ± 9.50 µSv, corresponding to 89.84% transmission and 10.16% shielding efficiency. The 0.5-mm lead reference reduced the dose to 251.34 µSv, with 19.03% transmission and 80.97% shielding efficiency. ML-BW50 again produced the lowest mean, at 228.42 µSv, corresponding to 17.30% transmission and 82.70% shielding efficiency. Compared with lead, ML-BW50 reduced the mean dose by approximately 9.12% and improved shielding efficiency by approximately 1.73 percentage points. These descriptive findings identify ML-BW50 as the highest-performing formulation at both ^99m^Tc activities.

The strong attenuation achieved by ML-BW50 may be associated with its alternating B50/W50/B50/W50 structure, which combines Bi_2_O_3_- and WO_3_-filled epoxy layers, with the Bi_2_O_3_-containing layer facing the radiation source. The high atomic numbers and densities of bismuth and tungsten increase the probability of interaction with the 140.5-keV photons emitted by ^99m^Tc, while the multilayer structure may provide sequential attenuation of primary and scattered photons. Overall, Table 2 supports ML-BW50 as a promising lead-free shielding formulation for ^99m^Tc radiation under the investigated experimental conditions.

At 925 MBq (25 mCi), NE did not significantly attenuate ^99m^Tc radiation relative to the no-shield control (Tukey-adjusted *p*-value 0.1897; Games–Howell-adjusted *p*-value 0.5860) (Table 3). In contrast, multilayer construction produced the strongest and most consistent shielding effect. Compared with their corresponding monolithic formulations, ML-BW25, ML-WB25, ML-BW50, and ML-WB50 reduced the dose equivalent by approximately 52.1%, 49.9%, 67.8%, and 68.5%, respectively; all comparisons were significant using both Tukey and Games–Howell tests, with adjusted *p*-values below 0.0001. ML-BW50 produced the lowest mean of 33.148 µSv, compared with 35.558 µSv for 0.5-mm lead. However, this 2.410 µSv difference was not statistically significant (Tukey *p*-value 0.9198; Games–Howell *p*-value 0.8077). Therefore, ML-BW50 showed numerical performance similar to lead.

Layer sequence also influenced attenuation. ML-BW25 produced a mean 5.184 µSv lower than ML-WB25, but its significance depended on the statistical method (Tukey *p*-value 0.0847; Games–Howell *p*-value 0.0013), making this finding inconclusive. At 50 phr, ML-BW50 produced an 18.1% lower dose than ML-WB50, and the difference was significant using both Tukey (*p*-value 0.0023) and Games–Howell (*p*-value 0.0335) tests. This provides stronger evidence that the Bi_2_O_3_-first configuration improved shielding at the higher filler loading. Overall, Table 3 identifies multilayer architecture as the dominant factor improving attenuation and ML-BW50 as the best-performing lead-free formulation at 925 MBq (25 mCi).

At 6.105 GBq (165 mCi), neat epoxy produced a significantly lower dose equivalent than the no-shield control (1186.45 versus 1320.60 µSv; mean difference 134.16 µSv; both adjusted *p*-values below 0.0001) (Table 4). Bi_2_O_3_ also provided greater attenuation than WO_3_ at matched loading: B25 was 48.51 µSv lower than W25 (Tukey *p*-value below 0.0001; Games–Howell *p*-value 0.0004), while B50 was 191.43 µSv lower than W50 (both adjusted *p*-values below 0.0001). More importantly, multilayer construction markedly outperformed the corresponding monolithic formulations. ML-BW25, ML-WB25, ML-BW50, and ML-WB50 reduced the dose by approximately 60.9%, 44.9%, 71.6%, and 64.6%, respectively, with all Tukey- and Games–Howell-adjusted *p*-values below 0.0001. This consistent agreement identifies multilayer architecture as the dominant factor improving the attenuation.

ML-BW50 was the best-performing formulation, producing 228.42 µSv compared with 251.34 µSv for 0.5-mm lead, representing a significant reduction of 22.92 µSv or 9.12% (Tukey *p*-value 0.0009; Games–Howell *p*-value 0.0276). In contrast, ML-WB50 produced 352.16 µSv and was significantly less effective than lead (both adjusted *p*-values below 0.0001). Layer sequence therefore had a pronounced effect: ML-BW25 and ML-BW50 produced approximately 32.3% and 35.1% lower dose than their corresponding WO_3_-first configurations, with both statistical methods giving *p*-values below 0.0001. Overall, Table 4 provides robust evidence that the Bi_2_O_3_-first multilayer architecture, particularly ML-BW50, delivered the greatest shielding performance at 6.105 GBq (165 mCi).

**Figure 8:**
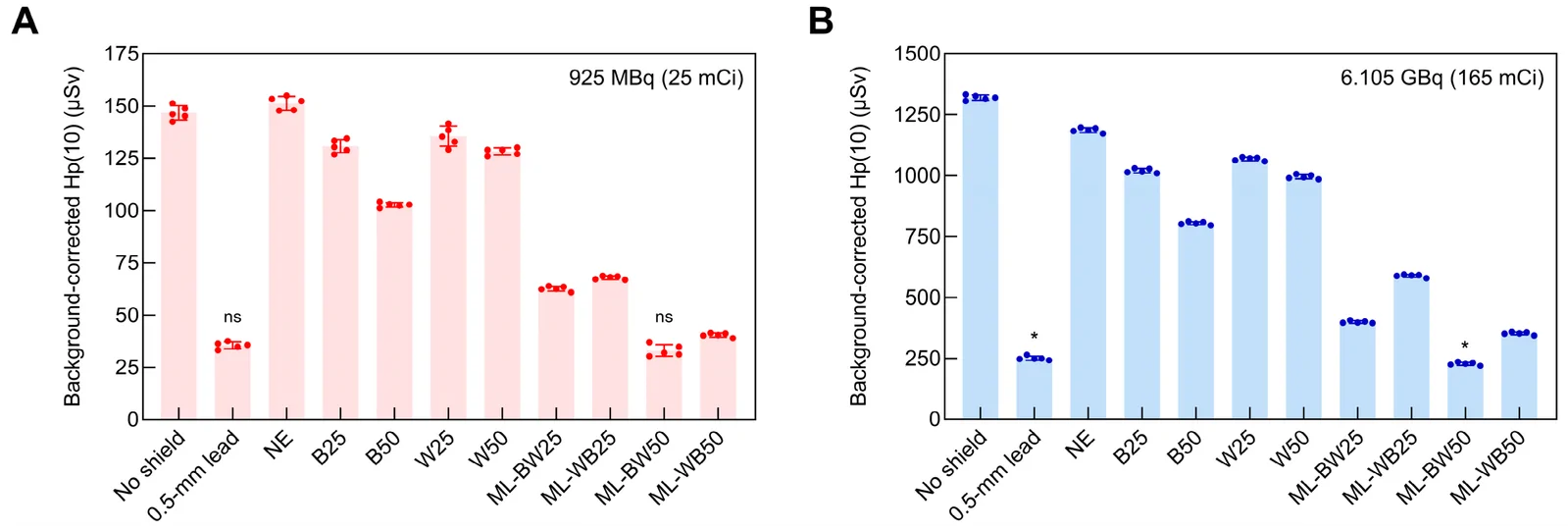
Background-corrected personal dose equivalent, Hp(10), measured for unshielded reference, 0.5-mm lead, neat epoxy resin (NE), monolithic Bi_2_O_3_- and WO_3_-filled epoxy composites, and hybrid multilayer composites during ^99m^Tc irradiation at (**A**) 925 MBq (25 mCi) and (**B**) 6.105 GBq (165 mCi). ML-BW50 denotes the B50/W50/B50/W50 multilayer sequence. Bars represent the mean ± SD (*n* = 5), and the superimposed colored points represent the five individual measurements obtained for each shielding condition. Light-pink bars with red points denote the 925 MBq condition in panel A, whereas light-blue bars with blue points denote the 6.105 GBq condition in panel B. Statistical annotations refer to the comparison between the 0.5-mm lead reference and ML-BW50; ns, *p* > 0.05; * *p* < 0.05.

Figure 8 demonstrates clear effects of nanoparticle type, filler loading, and multilayer architecture on the background-corrected personal dose equivalent. At both ^99m^Tc activity levels, increasing the nanoparticle loading from 25 to 50 phr generally reduced the measured dose, indicating improved photon attenuation at the higher filler concentration. Bi_2_O_3_-filled epoxy also generally produced a lower dose than WO_3_-filled epoxy at the same loading, particularly at 50 phr. This numerical advantage may be related to the higher atomic number of bismuth and its greater interaction probability with the 140.5-keV photons emitted by ^99m^Tc. Additionally, the multilayer composites produced substantially lower doses than the corresponding monolithic formulations at both activity levels. At 925 MBq (25 mCi), the mean doses were 62.69, 67.88, 33.15, and 40.47 µSv for ML-BW25, ML-WB25, ML-BW50, and ML-WB50, respectively. At 6.105 GBq (165 mCi), the corresponding values were 398.49, 588.16, 228.42, and 352.16 µSv. ML-BW50 produced the lowest mean dose in both panels of Figure 8 and was therefore identified as the best-performing composite. The Bi_2_O_3_-first configurations also consistently produced lower dose than the corresponding WO_3_-first configurations.

The comparison with 0.5-mm lead is presented in Figure 8 and quantified in Table 5. At 925 MBq, lead produced a mean dose of 35.56 ± 1.65 µSv, whereas ML-BW50 produced 33.15 ± 2.75 µSv. ML-BW50 therefore reduced the dose by 2.41 µSv, or 6.78%, relative to lead. Its relative radiation-shielding efficiency was also slightly higher than that of lead, with values of 77.42% and 75.77%, respectively. However, the difference was not statistically significant according to either Tukey’s test (adjusted *p*-value 0.9198) or the Games–Howell test (adjusted *p*-value 0.8077), consistent with the “ns” annotation in Figure 8A. Consequently, no significant difference between ML-BW50 and lead was detected at 925 MBq. However, at 6.105 GBq, lead produced a mean dose of 251.34 ± 8.28 µSv, whereas ML-BW50 produced 228.42 ± 6.50 µSv. This represented an absolute reduction of 22.92 µSv and a relative reduction of 9.12% compared with lead. ML-BW50 also increased the shielding efficiency from 80.97% to 82.70%, an improvement of 1.74 percentage points. The difference was statistically significant using both Tukey’s test (adjusted *p*-value 0.0009) and the Games–Howell test (adjusted *p*-value 0.0276), consistent with the asterisk in Figure 8B. Agreement between the two statistical approaches strengthens the conclusion that ML-BW50 produced a lower dose equivalent than lead under the 6.105-GBq experimental condition.

Although the statistical conclusions for the comparison between lead and ML-BW50 differed between the two activity levels, the absolute RSE advantage of ML-BW50 over lead remained similar, at 1.64 percentage points at 925 MBq and 1.74 percentage points at 6.105 GBq. Collectively, Figure 8 and Table 5 support ML-BW50 as the most effective lead-free formulation evaluated in this study. At 925 MBq, no statistically significant difference in personal dose equivalent was detected between ML-BW50 and 0.5-mm lead. At 6.105 GBq, ML-BW50 produced a significantly lower personal dose equivalent than the lead reference. These activity-specific findings support the shielding potential of ML-BW50 under the tested experimental conditions.

## 4. Discussion

The integrated physicochemical characterization and radiation-shielding measurements demonstrate that sequential casting generated an alternating, compositionally differentiated Bi- and W-containing epoxy architecture. Among the investigated lead-free formulations, the B50/W50/B50/W50 configuration (ML-BW50) consistently produced the lowest background-corrected personal dose equivalent, Hp(10), and the highest radiation-shielding efficiency at both ^99m^Tc activity levels. DLS and zeta-potential measurements characterized the hydrodynamic and electrokinetic behavior of the oxide dispersions; cross-sectional FE-SEM and EDX mapping established the cured-composite morphology and elemental distribution; XRD identified the crystalline phase assemblage; FTIR confirmed the principal chemical structure of the cured epoxy network; and contact-angle measurements characterized surface wettability. Considered together with filler loading, geometric bulk density, areal density, and layer sequence, these complementary results provide a coherent experimental basis for the enhanced attenuation achieved by ML-BW50 relative to the other lead-free formulations.

Dynamic light scattering analysis yielded Z-average hydrodynamic diameters of 646.41 nm for Bi_2_O_3_ and 413.38 nm for WO_3_, with corresponding cumulant polydispersity indices (PDIs) of 0.28 and 0.21, respectively (Figure 3). The Z-average for WO_3_ was therefore 233.03 nm (36.1%) lower than that for Bi_2_O_3_. The Z-average is an intensity-weighted, cumulant-derived equivalent-sphere diameter of the scattering entities undergoing translational diffusion in the liquid medium. It incorporates the inorganic particle core, the associated interfacial or solvation layer, and any particle aggregates or agglomerates present during measurement; consequently, these values characterize the hydrodynamic dispersion state and not the primary crystallite dimensions. Because the intensity of light scattered by small particles increases strongly with particle diameter—approximately in proportion to the sixth power of diameter within the Rayleigh-scattering regime—a relatively small number of larger particle associations can contribute disproportionately to the measured Z-average [47]. Additionally, the observation of Z-average diameters greater than the nominal 0.20 µm membrane-pore size is physically consistent with the principles of membrane filtration and DLS measurement. A nominal pore-size rating describes the filtration characteristics of the membrane and does not constitute an absolute hydrodynamic-diameter cutoff. Particle passage is influenced by the membrane pore-size distribution, particle geometry, minimum cross-sectional dimension, orientation during passage, particle–membrane interactions, and the dynamic association state of the dispersion. In addition, DLS converts measured translational-diffusion behavior into an equivalent spherical hydrodynamic diameter; therefore, irregular particles or post-filtration particle associations can produce hydrodynamic diameters exceeding the nominal membrane rating. The reported values consequently represent the scattering populations present in the filtered dispersions at the time of analysis. Under identical preparation and measurement conditions, the lower Z-average and PDI of WO_3_ indicate a smaller hydrodynamic population with a narrower apparent distribution of diffusion coefficients than that of Bi_2_O_3_. The zeta potentials of Bi_2_O_3_ and WO_3_ were −33.70 and −31.65 mV, respectively, with an absolute difference of only 2.05 mV, demonstrating comparable negatively charged particle–liquid interfaces. These magnitudes are consistent with an electrostatic contribution to dispersion stability under the applied pH, ionic-strength, and solvent conditions. The occurrence of a larger Bi_2_O_3_ Z-average despite its slightly greater absolute zeta-potential magnitude further indicates that the measured association state was governed collectively by particle surface chemistry, solvation, and interparticle interactions. Therefore, these liquid-phase measurements characterize the hydrodynamic and electrokinetic behavior of the synthesized powders under the specified DLS conditions. Their spatial distribution after incorporation into the cured epoxy matrix was evaluated separately by SEM imaging and EDS elemental mapping. Accordingly, the shielding behavior of the composites was considered in relation to filler composition, geometric bulk density, areal density, cured-composite morphology, elemental distribution, and multilayer architecture. Previous studies reporting improved attenuation in nano- or mixed micro/nanoparticle Bi_2_O_3_- and WO_3_-filled epoxy systems have associated the observed performance with improved filler distribution, reduced interparticle spacing, and lower void content, provided that extensive agglomeration is controlled [18,48,49].

The FE-SEM and EDX observations confirmed that the intended four-layer structure was retained after sequential casting (Figure 4). The complementary Bi- and W-rich bands verified the B50/W50/B50/W50 sequence, while the generally continuous interfaces indicate effective contact between adjacent layers within the examined cross-sectional regions. Localized agglomerates, pores, and particle-pull-out cavities were nevertheless observed, particularly near some interlayer boundaries. Such features are consistent with the high viscosity and reduced particle mobility expected at elevated inorganic-filler loadings. Previous studies of Bi_2_O_3_-filled epoxy systems have similarly shown that increasing filler concentration can intensify agglomeration and generate microstructural heterogeneity [40]. Although these defects could provide local low-density paths for photon transmission and potentially weaken the interfaces, the spatial separation of Bi- and W-rich regions was successfully maintained. Comparable multilayer shielding studies have reported that controlled distribution of high-atomic-number fillers across successive layers can improve attenuation relative to single-layer configurations, provided that void formation and filler segregation are adequately controlled [29].

The XRD patterns presented in Figure 5 revealed clear structural differences among the investigated materials. The 0.5-mm lead reference exhibited sharp diffraction peaks characteristic of crystalline Pb, whereas neat epoxy resin displayed a broad amorphous halo, consistent with its predominantly noncrystalline polymer structure. In ML-BW50, distinct crystalline reflections were superimposed on the amorphous epoxy background and were assigned to α-Bi_2_O_3_, tungsten trioxide (WO_3_), sodium tungstate dihydrate (Na_2_WO_4_·2H_2_O), and bismuth oxide carbonate hydroxide [(BiO)_4_CO_3_(OH)_2_]. The simultaneous identification of these Bi- and W-containing crystalline phases confirms the incorporation of both filler systems into ML-BW50 and demonstrates that crystalline inorganic phases were present within the cured epoxy composite. The coexistence of α-Bi_2_O_3_ with bismuth oxide carbonate hydroxide, together with WO_3_ and residual Na_2_WO_4_·2H_2_O, establishes a mixed crystalline phase assemblage produced under the applied precipitation, aging, and low-temperature drying conditions. In particular, the concurrent detection of WO_3_ and Na_2_WO_4_·2H_2_O indicates partial conversion of the sodium tungstate precursor. Comparable formation and thermal evolution of bismuth oxycarbonate phases and hydrated tungsten-containing intermediates during aqueous precipitation have previously been reported [41,46]. Accordingly, the measured radiation-shielding performance is reported for the complete ML-BW50 composite, which comprises the mixed Bi- and W-containing crystalline phase assemblage, its distribution within the epoxy matrix, and the alternating multilayer architecture.

In addition, the FTIR spectra showed that the principal chemical structure of the cured epoxy network was retained following filler incorporation and multilayer fabrication (Figure 6). The bands at approximately 3385 and 2928 cm^−1^ were assigned to hydroxyl/amine and aliphatic C–H stretching, respectively, whereas the bands at approximately 1609 and 1510 cm^−1^ originated from the aromatic rings of the DGEBA-based epoxy. The absorptions near 1238 and 1036 cm^−1^ were associated with ether-related C–O–C and C–O vibrations. Importantly, the low-wavenumber bands near 699 and 562 cm^−1^ were consistent with Bi–O and W–O lattice vibrations, providing spectroscopic evidence for the incorporation of the metal-oxide fillers into the epoxy composites. In ML-BW50, the presence of these metal–oxygen features together with the characteristic epoxy bands supports the successful integration of both Bi- and W-containing components without substantial disruption of the polymer backbone. When considered together with the XRD phase assignments and EDX elemental maps, the FTIR results confirm the presence of both filler systems within the ML-BW50 composite. These assignments agree with reported FTIR characteristics of amine-cured DGEBA epoxy systems [50]. Changes in band intensity, particularly near the oxirane-related band at approximately 916 cm^−1^, may indicate differences in residual epoxide content or local filler–polymer interactions; however, the absence of new dominant organic bands suggests that filler incorporation occurred primarily through physical and interfacial interactions rather than the formation of new covalent bonds.

Furthermore, the PBS contact-angle results support the potential practical suitability of ML-BW50 for ^99m^Tc handling environments in nuclear medicine (Figure 7). All apparent contact angles exceeded 90°, indicating relatively low wettability by PBS, which was used as a nonradioactive aqueous simulant of ^99m^Tc radiopharmaceutical solutions. ML-BW50 exhibited left- and right-side contact angles of 102.45° and 102.24°, respectively, exceeding those of neat epoxy (approximately 99.3°), B50 (97.8°), and W50 (92.8°). Consequently, PBS droplets remained more compact and spread less extensively over the ML-BW50 surface than over the monolithic composite surfaces. In clinical radiopharmacy settings, where accidental droplets may occur during ^99m^Tc preparation, dispensing, or administration, this reduced wetting could limit the initially contaminated surface area, facilitate visual identification and wiping, and reduce liquid penetration when combined with a continuous, low-porosity epoxy surface [51]. Together with its superior attenuation of 140.5-keV photons, these surface characteristics support ML-BW50 as a promising dual-function material for lead-free shielding components, work-surface inserts, syringe-preparation barriers, or protective panels used in ^99m^Tc clinical practice.

Comparatively, radiation-shielding performance was strongly dependent on filler type, loading, and specimen architecture (Figure 8 and Table 2, Table 3 and Table 4). Formulation had a significant effect on the background-corrected personal dose equivalent, Hp(10), at both 925 MBq and 6.105 GBq, with one-way ANOVA yielding F(10, 44) = 1656.48 and F(10, 44) = 13,703.69, respectively (both *p* < 0.0001). Increasing the filler concentration from 25 to 50 phr generally improved attenuation in the monolithic Bi_2_O_3_- and WO_3_-filled composites. This trend is consistent with a greater concentration of high-atomic-number photon-interaction sites and an increase in effective composite density at higher filler loadings. Previous investigations have similarly demonstrated that Bi_2_O_3_- and WO_3_-containing epoxy composites enhance attenuation within the energy range relevant to ^99m^Tc, with performance influenced by filler concentration, particle size, density, and dispersion [15,18,26]. Furthermore, the multilayer specimens produced substantially lower dose equivalent than their corresponding monolithic formulations, with reductions of approximately 45–72%, depending on filler loading and source activity. ML-BW50 produced the lowest at both activities: 33.15 ± 2.75 µSv at 925 MBq and 228.42 ± 6.50 µSv at 6.105 GBq. The corresponding radiation-shielding efficiencies were 77.42% and 82.70%, respectively, identifying ML-BW50 as the best-performing lead-free formulation evaluated. Layer sequence also influenced attenuation, particularly at 6.105 GBq. The Bi_2_O_3_-first ML-BW50 sequence produced a lower dose equivalent than the reverse ML-WB50 sequence at both activities, whereas the statistical significance of the ML-BW25 versus ML-WB25 comparison at 925 MBq depended on the post hoc method applied.

Consequently, comparison with the 0.5-mm lead reference provided the most clinically relevant finding (Table 5). At 925 MBq, lead and ML-BW50 produced dose equivalents of 35.56 ± 1.65 and 33.15 ± 2.75 µSv, respectively. No statistically significant difference was detected using either Tukey’s or Games–Howell testing, indicating that no performance difference was detected between the two materials under the 925-MBq condition. At 6.105 GBq, ML-BW50 produced a significantly lower dose equivalent than lead—228.42 ± 6.50 versus 251.34 ± 8.28 µSv—according to both Tukey-adjusted (*p* = 0.0009) and Games–Howell-adjusted (*p* = 0.0276) comparisons. ML-BW50 therefore reduced the measured dose equivalent by 9.12% relative to lead under this condition. Despite the different statistical outcomes, ML-BW50 exhibited a similar radiation-shielding-efficiency advantage over lead at both activities: 1.64 percentage points at 925 MBq and 1.74 percentage points at 6.105 GBq.

In summary, the results identify ML-BW50 as the most promising lead-free shielding formulation investigated. It combined the lowest measured ^99m^Tc dose equivalent, a retained multilayer architecture, and relatively low wettability by PBS. The tested ML-BW50 specimen was also approximately 42.9% lighter than the tested lead reference.

## 5. Conclusions

This study fabricated monolithic Bi_2_O_3_–epoxy and WO_3_–epoxy composites and four-layer hybrid Bi_2_O_3_/WO_3_–epoxy composites and evaluated their attenuation of 140.5-keV photons emitted by ^99m^Tc. DLS analysis produced Z-average hydrodynamic diameters of 646.41 and 413.38 nm and cumulant polydispersity indices of 0.28 and 0.21 for the Bi_2_O_3_ and WO_3_ dispersions, respectively. Their corresponding zeta potentials of −33.70 and −31.65 mV established negatively charged particle–liquid interfaces under the applied measurement conditions. Cross-sectional FE-SEM imaging and EDX elemental mapping documented retention of the alternating B50/W50/B50/W50 architecture and compositionally differentiated Bi-rich and W-rich layers within ML-BW50. XRD identified α-Bi_2_O_3_, bismuth oxide carbonate hydroxide, WO_3_, and Na_2_WO_4_·2H_2_O within the amorphous epoxy matrix, establishing a mixed-phase Bi- and W-containing filler system and partial conversion of the sodium tungstate precursor. FTIR spectra retained the characteristic bands of the cured epoxy network together with filler-associated spectral features. ML-BW50 exhibited PBS contact angles of 102.45° and 102.24° on the left and right measurement sides, respectively, suggesting potential advantages for limiting the initial spreading of aqueous radiopharmaceutical droplets. Additionally, supplementary tensile characterization showed that ML-BW50 achieved a maximum tensile stress of 4.008 MPa, a terminal strain of 74.294%, and a tensile toughness of 2.250 MJ·m^−3^. Its maximum tensile stress exceeded those of B50 and W50 by 7.7% and 38.3%, respectively, while its terminal strain was 18.3% and 14.9% higher. The tensile toughness of ML-BW50 exceeded those of NE, B50, and W50 by 4.0%, 16.7%, and 43.2%, respectively, representing the highest tensile-energy absorption among the investigated epoxy-based formulations. These results demonstrate that the alternating multilayer architecture combined improved tensile strength, deformation capacity, and energy absorption relative to the corresponding high-loading monolithic composites.

Moreover, the shielding formulation produced a significant overall effect on the background-corrected personal dose equivalent, Hp(10), at both 925 MBq (25 mCi) and 6.105 GBq (165 mCi) (one-way ANOVA, both *p* < 0.0001). Increasing filler loading from 25 to 50 phr generally improved attenuation, while the multilayer composites reduced the dose equivalent by approximately 45–72% relative to their corresponding monolithic formulations. ML-BW50 was the most effective lead-free formulation and produced mean Hp(10) values of 33.15 ± 2.75 and 228.42 ± 6.50 µSv, corresponding to radiation-shielding efficiencies of 77.42% and 82.70% at 925 MBq and 6.105 GBq, respectively. The post hoc comparison at 925 MBq identified no statistically significant difference between ML-BW50 and the 0.5-mm lead reference. At 6.105 GBq, ML-BW50 produced a significantly lower Hp(10) than the lead reference, corresponding to a relative dose reduction of 9.12%. For the same projected area of 10 × 10 cm, the ML-BW50 specimen had a mass of 44.0 g compared with 77.0 g for the lead reference, representing a 42.9% reduction in specimen mass. Collectively, ML-BW50 provided the strongest ^99m^Tc attenuation among the investigated lead-free formulations while combining lower specimen mass, an organized multilayer structure, low PBS wettability, and the highest tensile toughness among the epoxy-based materials. These combined characteristics support the continued development of hybrid Bi_2_O_3_/WO_3_ multilayer epoxy composites as lead-free shielding materials for occupational radiation protection in nuclear medicine.

Further development will extend the experimental evaluation across photon energies associated with other clinically important diagnostic and therapeutic radionuclides and incorporate a larger number of independently fabricated specimens. Direct radiopharmaceutical spill-recovery and decontamination studies will quantify surface cleanability under operational conditions. Interlayer adhesion, long-term aging, elemental leaching, and resistance to repeated cleaning cycles will provide additional evidence concerning structural durability and service performance. Together, these investigations will establish the energy-dependent attenuation, fabrication reproducibility, decontamination behavior, and long-term functional stability of the multilayer composite system.

## Figures and Tables

**Table 1:** Formulations, architectures, dimensions, masses, geometric bulk densities, and areal densities of the shielding specimens.

Specimen ^1^ (Code)	Architecture and Filler/Layer Sequence, Source → Detector	Nominal Filler Loading ^2^ (phr)	Filler Loading (phr)	Mass (g)	Dimensions ^3^, L × W × t (cm)	Volume (cm^3^)	Geometric Bulk Density ^4^ (g·cm^−3^)	Areal Density ^5^ (g·cm^−2^)
0.5-mm lead reference (Pb)	Single sheet	—	—	77.0	10 × 10 × 0.05	5	15.400	0.77
Neat epoxy resin (NE)	Monolithic; no filler	0	0	19.0	10 × 10 × 2	200	0.095	0.19
Bi-containing-powder/epoxy (B25)	Monolithic; Bi-containing powder	25	25	26.0	10 × 10 × 2	200	0.130	0.26
Bi-containing-powder/epoxy (B50)	Monolithic; Bi-containing powder	50	50	38.0	10 × 10 × 2	200	0.190	0.38
W-containing-powder/epoxy (W25)	Monolithic; W-containing powder	25	25	26.0	10 × 10 × 2	200	0.130	0.26
W-containing-powder/epoxy (W50)	Monolithic; W-containing powder	50	50	38.0	10 × 10 × 2	200	0.190	0.38
Hybrid multilayer BW25 (ML-BW25)	B25/W25/B25/W25	25 per filled layer	25	29.0	10 × 10 × 2	200	0.145	0.29
Hybrid multilayer WB25 (ML-WB25)	W25/B25/W25/B25	25 per filled layer	25	29.0	10 × 10 × 2	200	0.145	0.29
Hybrid multilayer BW50 (ML-BW50)	B50/W50/B50/W50	50 per filled layer	50	44.0	10 × 10 × 2	200	0.220	0.44
Hybrid multilayer WB50 (ML-WB50)	W50/B50/W50/B50	50 per filled layer	50	44.0	10 × 10 × 2	200	0.220	0.44

^1^ Phase-code footnote: B and W are operational formulation codes denoting layers containing the synthesized Bi-containing and W-containing powders, respectively. XRD analysis of ML-BW50 identified α-Bi_2_O_3_ and (BiO)_4_CO_3_(OH)_2_ among the Bi-containing crystalline phases and WO_3_ and Na_2_WO_4_·2H_2_O among the W-containing crystalline phases. ^2^ Filler loading is expressed in parts per hundred parts of epoxy resin by mass (phr), corresponding to the mass of synthesized filler powder added per 100 parts by mass of epoxy resin before curing-agent addition. The epoxy resin-to-curing-agent mass ratio was 3:1 in every epoxy formulation. ^3^ The dimensions represent the complete specimens used for radiation measurements. For each four-layer specimen, the reported 2.0-cm value represents the total assembly thickness, corresponding to a nominal thickness of approximately 0.50 cm per layer. ^4^ Geometric bulk density was calculated as $\rho_{g}=\;\frac{m}{V}$, where m is the complete specimen mass and V = L × W × t is its external volume. ^5^ Areal density perpendicular to the incident photon beam was calculated as $\sigma =\frac{m}{A}$, where A = L × W = 100.0 cm^2^. Equivalently, $\sigma =\rho_{g}\times t$.

**Table 2:** Activity-specific background-corrected personal dose equivalent, radiation transmission, and relative radiation-shielding efficiency (RSE) of neat epoxy resin, 0.5-mm lead, and the ML-BW50 composite during ^99m^Tc irradiation at nominal activities of 925 MBq (25 mCi) and 6.105 GBq (165 mCi). ML-BW50 denotes the four-layer sequence B50/W50/B50/W50, with the Bi_2_O_3_-containing layer facing the source. The measured endpoint was the personal dose equivalent, Hp(10). Values are presented as mean ± SD (*n* = 5).

^99m^Tc Activity	Condition	Hp(10) (µSv), Mean ± SD	Transmission (%)	RSE (%)
925 MBq (25 mCi)	No shield	146.77 ± 3.45	100.00	0.00
	NE	151.35 ± 3.33	103.12	−3.12
	0.5-mm lead	35.56 ± 1.65	24.23	75.77
	ML-BW50	33.15 ± 2.75	22.58	77.42
6.105 GBq (165 mCi)	No shield	1320.60 ± 10.32	100.00	0.00
	NE	1186.45 ± 9.50	89.84	10.16
	0.5-mm lead	251.34 ± 8.28	19.03	80.97
	ML-BW50	228.42 ± 6.50	17.30	82.70

**Table 3:** Selected pairwise comparisons of background-corrected personal dose equivalent, Hp(10), measured during ^99m^Tc irradiation at 925 MBq (25 mCi).

Comparison	Group 1 Mean (µSv)	Group 2 Mean (µSv)	Mean Difference * (µSv)	Tukey- Adjusted *p*	Games–Howell- Adjusted *p*	Interpretation
No shield vs. NE	146.770	151.349	−4.579	0.1897	0.5860	No statistically significant attenuation by NE
0.5-mm lead vs. ML-BW50	35.558	33.148	2.410	0.9198	0.8077	No statistically significant difference detected
0.5-mm lead vs. ML-WB50	35.558	40.465	−4.907	0.1244	0.0164	Significance depended on the post hoc method
B25 vs. ML-BW25	130.893	62.692	68.201	<0.0001	<0.0001	The multilayer architecture significantly reduced the dose equivalent
W25 vs. ML-WB25	135.554	67.876	67.678	<0.0001	<0.0001	The multilayer architecture significantly reduced the dose equivalent
B50 vs. ML-BW50	102.817	33.148	69.669	<0.0001	<0.0001	The multilayer architecture significantly reduced the dose equivalent
W50 vs. ML-WB50	128.334	40.465	87.869	<0.0001	<0.0001	The multilayer architecture significantly reduced the dose equivalent
ML-BW25 vs. ML-WB25	62.692	67.876	−5.184	0.0847	0.0013	The Bi_2_O_3_-first sequence had a lower mean; significance was method-dependent
ML-BW50 vs. ML-WB50	33.148	40.465	−7.317	0.0023	0.0335	The Bi_2_O_3_-first sequence produced a significantly lower dose equivalent

* Mean difference was calculated as Group 1 minus Group 2. B25 and B50 denote Bi_2_O_3_-filled epoxy layers containing 25 and 50 phr filler, respectively; W25 and W50 denote the corresponding WO_3_-filled layers. Tukey’s test followed ordinary one-way ANOVA, whereas the Games–Howell test was used following Welch’s ANOVA. Statistical significance was defined as *p* < 0.05. Values calculated as 0.0000 are appropriately reported as *p* < 0.0001.

**Table 4:** Selected pairwise comparisons of background-corrected personal dose equivalent, Hp(10), measured during ^99m^Tc irradiation at 6.105 GBq (165 mCi).

Comparison	Group 1 Mean (µSv)	Group 2 Mean (µSv)	Mean Difference * (µSv)	Tukey- Adjusted *p*	Games–Howell- Adjusted *p*	Interpretation
No shield vs. NE	1320.60	1186.45	134.16	<0.0001	<0.0001	The neat epoxy condition had a lower mean than the unshielded reference at 165 mCi.
0.5-mm lead vs. ML-BW50	251.34	228.42	22.92	0.0009	0.0276	The Bi_2_O_3_-first 50-phr multilayer had a lower mean dose equivalent than the lead reference.
0.5-mm lead vs. ML-WB50	251.34	352.16	−100.82	<0.0001	<0.0001	The 0.5-mm lead reference had a lower mean dose equivalent than the WO_3_-first 50-phr multilayer.
B25 vs. W25	1019.22	1067.73	−48.51	<0.0001	0.0004	At 25 phr, the Bi_2_O_3_ monolithic composite had the lower mean dose equivalent.
B50 vs. W50	803.85	995.28	−191.43	<0.0001	<0.0001	At 50 phr, the Bi_2_O_3_ monolithic composite had the lower mean dose equivalent.
B25 vs. ML-BW25	1019.22	398.49	620.73	<0.0001	<0.0001	The alternating multilayer significantly reduced dose equivalent relative to the matched B25 monolithic composite.
W25 vs. ML-WB25	1067.73	588.16	479.56	<0.0001	<0.0001	The alternating multilayer significantly reduced the dose equivalent relative to the matched W25 monolithic composite.
B50 vs. ML-BW50	803.85	228.42	575.43	<0.0001	<0.0001	The alternating multilayer significantly reduced dose equivalent relative to the matched B50 monolithic composite.
W50 vs. ML-WB50	995.28	352.16	643.12	<0.0001	<0.0001	The alternating multilayer significantly reduced dose equivalent relative to the matched W50 monolithic composite.
ML-BW25 vs. ML-WB25	398.49	588.16	−189.67	<0.0001	<0.0001	The Bi_2_O_3_-first 25-phr sequence produced the lower dose equivalent.
ML-BW50 vs. ML-WB50	228.42	352.16	−123.74	<0.0001	<0.0001	The Bi_2_O_3_-first 50-phr sequence produced the lower dose equivalent.

* Tukey HSD is the primary post hoc method after ordinary one-way ANOVA. Games–Howell is shown as a heteroscedasticity sensitivity analysis. Values shown as <0.0001 were below the reporting threshold.

**Table 5:** Side-by-side statistical comparison of the 0.5-mm lead reference and the ML-BW50 hybrid multilayer epoxy composite during ^99m^Tc irradiation at nominal activities of 925 MBq (25 mCi) and 6.105 GBq (165 mCi). ML-BW50 denotes the B50/W50/B50/W50 multilayer sequence. Values of Hp(10) are background-corrected means ± SD (*n* = 5). RSE was calculated relative to the no-shield control at the corresponding activity. The dose difference was calculated as lead minus ML-BW50, and the RSE difference as ML-BW50 minus lead. Tukey’s and Games–Howell’s *p*-values are multiplicity-adjusted; statistical significance was defined as *p* < 0.05.

Parameter	^99m^Tc 925 MBq (25 mCi)	^99m^Tc 6.105 GBq (165 mCi)
0.5-mm lead, Hp(10) (µSv)	35.56 ± 1.65	251.34 ± 8.28
ML-BW50, Hp(10) (µSv)	33.15 ± 2.75	228.42 ± 6.50
0.5-mm lead RSE (%)	75.77	80.97
ML-BW50 RSE (%)	77.42	82.70
RSE difference, ML-BW50 − lead (percentage points)	+1.64	+1.74
Dose difference, lead − ML-BW50 (µSv)	2.41	22.92
ML-BW50 dose reduction relative to lead (%)	6.78	9.12
Tukey-adjusted *p*-value	0.9198	0.0009
Games–Howell-adjusted *p*-value	0.8077	0.0276
Interpretation	No statistically significant difference detected	ML-BW50 produced a significantly lower dose equivalent

## Data Availability

The original contributions presented in this study are included in the article material. Further inquiries can be directed to the corresponding author.

## Supplementary Materials

The following supporting information can be downloaded at https://www.mdpi.com/article/10.3390/polym18192434/s1. Figure S1: Comparative field-emission scanning electron microscopy (FE-SEM) micrographs of the 0.5-mm lead reference (Pb), neat epoxy resin (NE), 50-phr Bi_2_O_3_–epoxy monolithic composite (B50), and 50-phr WO_3_–epoxy monolithic composite (W50). Figure S2: Tensile mechanical behavior of the 0.5-mm lead reference and selected epoxy-based shielding materials. Table S1: Curve-derived tensile properties and tensile toughness of the 0.5-mm lead reference, neat epoxy, monolithic nanoparticle-filled composites, and the ML-BW50 hybrid multilayer composite.

## References

[1] International Atomic Energy Agency, Food and Agriculture Organization of the United Nations, World Health Organization (2022). Exposure Due to Radionuclides in Food Other Than During a Nuclear or Radiological Emergency. Part 2: Considerations in Implementing Requirement 51 of IAEA General Safety Requirements Part 3 (International Basic Safety Standards).

[2] Getaldić A., Surić Mihić M., Uroić G., Veinović Ž. (2023). Nuclear and Radiological Data Available in the International Atomic Energy Agency (IAEA) Databases. Rud. Geol. Naft. Zb..

[3] Pinak M., Joksic J., Vandenhove H. (2026). IAEA Safety Standards–One Step Toward Harmonized System of Radiation Protection Applied in Practice. Health Phys..

[4] Valentin J. (2008). The 2007 Recommendations of the International Commission on Radiological Protection.

[5] Marshall S.K., Prom-On P., Sangkue S., Thiangsook W. (2023). Assessment of Radiation Exposure in a Nuclear Medicine Department during ^99m^Tc-MDP Bone Scintigraphy. Toxics.

[6] Amako K., Guatelli S., Ivanchenko V.N., Maire M., Mascialino B., Murakami K., Nieminen P., Pandola L., Parlati S., Pia M.G. (2005). Comparison of Geant4 Electromagnetic Physics Models against the NIST Reference Data. IEEE Trans. Nucl. Sci..

[7] Berger M.J., Hubbell J.H. (1987). XCOM: Photon Cross Sections on a Personal Computer.

[8] Hubbell J. (1969). Photon Cross Sections, Attenuation Coefficients and Energy Absorption Coefficients.

[9] Chang Q., Guo S., Zhang X. (2023). Radiation Shielding Polymer Composites: Ray-Interaction Mechanism, Structural Design, Manufacture and Biomedical Applications. Mater. Des..

[10] More C.V., Alsayed Z., Badawi M.S., Thabet A.A., Pawar P.P. (2021). Polymeric Composite Materials for Radiation Shielding: A Review. Environ. Chem. Lett..

[11] Gilys L., Griškonis E., Griškevičius P., Adlienė D. (2022). Lead Free Multilayered Polymer Composites for Radiation Shielding. Polymers.

[12] Aldhuhaibat M.J., Amana M.S., Jubier N.J., Salim A. (2021). Improved Gamma Radiation Shielding Traits of Epoxy Composites: Evaluation of Mass Attenuation Coefficient, Effective Atomic and Electron Number. Radiat. Phys. Chem..

[13] Elsafi M., Hedaya A.M., Abdel-Gawad E.H., Rashad M., Sayyed M., Saleh I.H. (2024). Experimental Investigation of the Radiation Shielding Performance of a Newly Developed Silicon-Epoxy Resin Doped with WO_3_ Micro/Nanoparticles. Silicon.

[14] Verdipoor K., Alemi A., Mesbahi A. (2018). Photon Mass Attenuation Coefficients of a Silicon Resin Loaded with WO_3_, PbO, and Bi_2_O_3_ Micro and Nano-Particles for Radiation Shielding. Radiat. Phys. Chem..

[15] Karabul Y., İçelli O. (2021). The Assessment of Usage of Epoxy Based Micro and Nano-Structured Composites Enriched with Bi_2_O_3_ and WO_3_ Particles for Radiation Shielding. Results Phys..

[16] Molina Higgins M.C., Radcliffe N.A., Toro-González M., Rojas J.V. (2019). Gamma Ray Attenuation of Hafnium Dioxide-and Tungsten Trioxide-Epoxy Resin Composites. J. Radioanal. Nucl. Chem..

[17] Muthamma M., Prabhu S., Bubbly S., Gudennavar S. (2021). Micro and Nano Bi_2_O_3_ Filled Epoxy Composites: Thermal, Mechanical and γ-Ray Attenuation Properties. Appl. Radiat. Isot..

[18] Hedaya A., Elsafi M., Al-Saleh W.M., Saleh I.H. (2024). Effect of Bi_2_O_3_ Particle Size on the Radiation-Shielding Performance of Free-Lead Epoxide Materials against Ionizing Radiation. Polymers.

[19] Elsafi M., Almuqrin A.H., Almutairi H.M., Al-Saleh W.M., Sayyed M. (2023). Grafting Red Clay with Bi_2_O_3_ Nanoparticles into Epoxy Resin for Gamma-Ray Shielding Applications. Sci. Rep..

[20] Elsafi M., Almousa N., Al-Harbi N., Almutiri M., Yasmin S., Sayyed M. (2023). Ecofriendly and Radiation Shielding Properties of Newly Developed Epoxy with Waste Marble and WO_3_ Nanoparticles. J. Mater. Res. Technol..

[21] Marshall S.K., Chuaymuang J., Kwandee P., Songphum N. (2025). Shielding Efficacy of Tungsten Oxide-Reinforced Polyisoprene in Attenuating Technetium-99m Gamma Radiation: An Alternative Shielding Solution for Occupational Safety in Nuclear Medicine. Appl. Sci..

[22] Wu Z., Li Y., Yan Q., Liu G., Liu Y., Wang G., He L. (2022). Gamma Radiation Shielding Properties of WO_3_/Bi_2_O_3_/Waterborne Polyurethane Composites. J. Korean Phys. Soc..

[23] Chai H., Tang X., Ni M., Chen F., Zhang Y., Chen D., Qiu Y. (2016). Preparation and Properties of Novel, Flexible, Lead-free X-ray-shielding Materials Containing Tungsten and Bismuth (III) Oxide. J. Appl. Polym. Sci..

[24] Wang B., Qiu T., Yuan L., Fang Q., Wang X., Guo X., Zhang D., Lai C., Wang Q., Liu Y. (2023). A Comparative Study between Pure Bismuth/Tungsten and the Bismuth Tungsten Oxide for Flexible Shielding of Gamma/X Rays. Radiat. Phys. Chem..

[25] Zhang T., Li Y., Yuan Y., Cui K., Wei W., Wu J., Qin W., Wu X. (2022). Spatially Confined Bi_2_O_3_–Ti_3_C_2_Tx Hybrids Reinforced Epoxy Composites for Gamma Radiation Shielding. Compos. Commun..

[26] Marshall S.K., Tepnarin P., Wattanaphonpinich W., Atsawasetthini W. (2026). Experimental and Computational Evaluation of Hybrid Bi_2_O_3_/WO_3_ Nanoparticle-Filled Epoxy Composites for Lead-Free Tc-99m Gamma-Ray Shielding in Occupational Radiation Protection. Polymers.

[27] Li Z., Zhou W., Zhang X., Gao Y., Guo S. (2021). High-Efficiency, Flexibility and Lead-Free X-Ray Shielding Multilayered Polymer Composites: Layered Structure Design and Shielding Mechanism. Sci. Rep..

[28] Kim Y., Park S., Seo Y. (2015). Enhanced X-ray Shielding Ability of Polymer–Nonleaded Metal Composites by Multilayer Structuring. Ind. Eng. Chem. Res..

[29] Al-Saleh W.M., Almutairi H.M., Sayyed M., Elsafi M. (2023). Multilayer Radiation Shielding System with Advanced Composites Containing Heavy Metal Oxide Nanoparticles: A Free-Lead Solution. Sci. Rep..

[30] Ehlers J.-E., Rondan N.G., Huynh L.K., Pham H., Marks M., Truong T.N. (2007). Theoretical Study on Mechanisms of the Epoxy− Amine Curing Reaction. Macromolecules.

[31] Santiago D., Fernández-Francos X., Ramis X., Salla J.M., Sangermano M. (2011). Comparative Curing Kinetics and Thermal–Mechanical Properties of DGEBA Thermosets Cured with a Hyperbranched Poly (Ethyleneimine) and an Aliphatic Triamine. Thermochim. Acta.

[32] Perrin F., Nguyen M.H., Vernet J. (2009). Water Transport in Epoxy–Aliphatic Amine Networks–Influence of Curing Cycles. Eur. Polym. J..

[33] Li K., Wang K., Zhan M., Xu W. (2013). The Change of Thermal–Mechanical Properties and Chemical Structure of Ambient Cured DGEBA/TEPA under Accelerated Thermo-Oxidative Aging. Polym. Degrad. Stab..

[34] Tofanica B.-M., Ungureanu E., Awaja F. (2025). Surface Chemistry and Molecular Dynamics of Epoxy Resin: Insights from Analysis During Curing and Post-Curing Processes. Polymers.

[35] Farkas N., Kramar J.A. (2021). Dynamic Light Scattering Distributions by Any Means. J. Nanoparticle Res..

[36] Yeap S.P., Lim J., Ngang H.P., Ooi B.S., Ahmad A.L. (2018). Role of Particle–Particle Interaction towards Effective Interpretation of Z-Average and Particle Size Distributions from Dynamic Light Scattering (DLS) Analysis. J. Nanosci. Nanotechnol..

[37] Ullmann C., Babick F., Stintz M. (2019). Microfiltration of Submicron-Sized and Nano-Sized Suspensions for Particle Size Determination by Dynamic Light Scattering. Nanomaterials.

[38] Murdock R.C., Braydich-Stolle L., Schrand A.M., Schlager J.J., Hussain S.M. (2008). Characterization of Nanomaterial Dispersion in Solution Prior to in Vitro Exposure Using Dynamic Light Scattering Technique. Toxicol. Sci..

[39] Mahl D., Diendorf J., Meyer-Zaika W., Epple M. (2011). Possibilities and Limitations of Different Analytical Methods for the Size Determination of a Bimodal Dispersion of Metallic Nanoparticles. Colloids Surf. A Physicochem. Eng. Asp..

[40] Li W., Peng M., Zhao X., Chen S. (2022). Properties of Bi_2_O_3_/Epoxy Resin–Coated Composites for Protection against Gamma Rays. J. Ind. Text..

[41] Supothina S., Seeharaj P., Yoriya S., Sriyudthsak M. (2007). Synthesis of Tungsten Oxide Nanoparticles by Acid Precipitation Method. Ceram. Int..

[42] Ahmadi M., Younesi R., Guinel M.J.-F. (2014). Synthesis of Tungsten Oxide Nanoparticles Using a Hydrothermal Method at Ambient Pressure. J. Mater. Res..

[43] Neethu R., Madhu G. (2025). Influence of Oxalic Acid and CTAB on the Structural and Optical Properties of Nanocrystalline Tungsten Oxide Synthesized via Co-Precipitation Method. J. Mater. Sci. Mater. Eng..

[44] Moré R., Olah M., Balaghi S.E., Jäker P., Siol S., Zhou Y., Patzke G.R. (2017). Bi_2_O_2_CO_3_ Growth at Room Temperature: In Situ X-ray Diffraction Monitoring and Thermal Behavior. ACS Omega.

[45] Camilleri J., Borg J., Damidot D., Salvadori E., Pilecki P., Zaslansky P., Darvell B.W. (2020). Colour and Chemical Stability of Bismuth Oxide in Dental Materials with Solutions Used in Routine Clinical Practice. PLoS ONE.

[46] Valencia K., López A., Hernández-Gordillo A., Zanella R., Rodil S.E. (2018). Stabilized β-Bi_2_O_3_ Nanoparticles from (BiO)_4_CO_3_ (OH)_2_ Precursor and Their Photocatalytic Properties under Blue Light. Ceram. Int..

[47] Pochapski D.J., Carvalho dos Santos C., Leite G.W., Pulcinelli S.H., Santilli C.V. (2021). Zeta Potential and Colloidal Stability Predictions for Inorganic Nanoparticle Dispersions: Effects of Experimental Conditions and Electrokinetic Models on the Interpretation of Results. Langmuir.

[48] Azman N.N., Siddiqui S., Low I.M. (2013). Characterisation of Micro-Sized and Nano-Sized Tungsten Oxide-Epoxy Composites for Radiation Shielding of Diagnostic X-Rays. Mater. Sci. Eng. C.

[49] Marshall S.K., Boonpeng K., Buapud N., Chimhashat S., Chuaymuang J., Kwandee P., Songphum N. (2025). Bismuth Oxide Nanoparticle-Enhanced Poly (Methyl Methacrylate) Composites for I-131 Radiation Shielding: A Combined Simulation and Experimental Investigation. Polymers.

[50] Nikolic G., Zlatkovic S., Cakic M., Cakic S., Lacnjevac C., Rajic Z. (2010). Fast Fourier Transform IR Characterization of Epoxy GY Systems Crosslinked with Aliphatic and Cycloaliphatic EH Polyamine Adducts. Sensors.

[51] Ruhman N., Grantham V., Martin C. (2010). The Effectiveness of Decontamination Products in the Nuclear Medicine Department. J. Nucl. Med. Technol..

