# Supplementary material for: Hybrid Multilayer Epoxy Composites Filled with Mixed-Phase Bismuth- and Tungsten-Containing Nanoparticles for Lead-Free 99mTc Shielding in Occupational Nuclear Medicine
Source: Polymers (Basel). 2026 Oct 6;18(19):2434. doi: 10.3390/polym18192434 (PMC13666792; doi:10.3390/polym18192434)
Supplement: Supplementary file 1 [file polymers-18-02434-s001.zip › polymers-4576181-supplementary.pdf]

polymers

S1. FE-SEM Morphology of Reference and Monolithic Shielding Specimens

Supplementary Figure S1 presents representative FE-SEM micrographs of the 0.5-mm lead reference (Pb), neat epoxy resin (NE), 50-phr Bi_2O_3–epoxy composite (B50), and 50-phr WO_3–epoxy composite (W50) at the same nominal magnification. The Pb reference exhibited a comparatively compact surface with directional striations and localized surface irregularities. NE displayed a relatively continuous and fine-textured morphology without the conspicuous discrete particulate features observed in the filled composites.

Incorporation of the oxide fillers produced distinct morphological changes in the cured epoxy matrix. Within the examined field, B50 contained numerous rounded and irregular particulate features, including localized larger clusters and surface discontinuities. W50 exhibited a greater population of finer particulate features distributed across the examined region, together with occasional larger clusters. The observed differences demonstrate that the Bi- and W-containing fillers produced distinguishable cured-composite morphologies at the same nominal loading of 50 phr. The finer particulate morphology observed in W50 is also qualitatively consistent with the lower Z-average hydrodynamic diameter and polydispersity index measured for the WO_3 dispersion compared with the Bi_2O_3 dispersion.

B50 and W50 constitute the individual high-loading formulations used to fabricate the alternating B50/W50/B50/W50 layers of ML-BW50. Consequently, the supplementary micrographs provide direct morphological comparisons among the metallic reference, unfilled polymer matrix, and single-filler constituents of the hybrid multilayer composite. These FE-SEM observations complement the EDX elemental maps of ML-BW50, which document the distributions of C, O, Bi, and W signals within the analyzed region of the highest-performing composite.

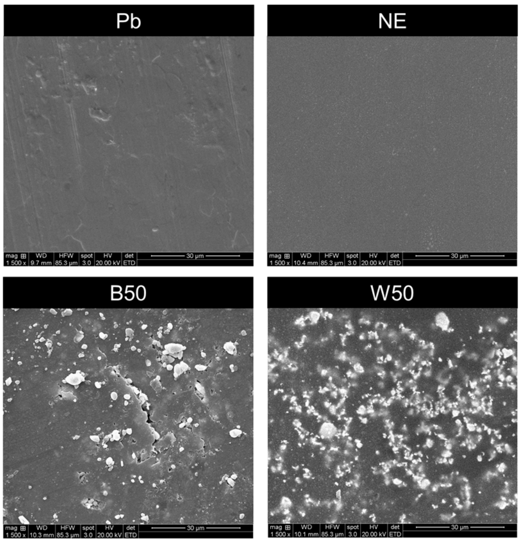

Figure S1. Comparative field-emission scanning electron microscopy (FE-SEM) micrographs of the 0.5-mm lead reference (Pb), neat epoxy resin (NE), 50-phr Bi_2O_3–epoxy monolithic composite (B50), and 50-phr WO_3–epoxy monolithic composite (W50). Images were acquired at a nominal magnification of 1500× and an accelerating voltage of 20.0 kV using a secondary-electron detector. Scale bars = 30 μm. B50 and W50 denote epoxy composites containing 50 parts of Bi_2O_3 or WO_3 filler, respectively, per 100 parts by mass of epoxy resin before curing-agent addition; these formulations constitute the individual filled layers of the ML-BW50 hybrid composite.

S2. Tensile Stress–Strain Behavior and Derived Mechanical Properties of the Selected Shielding Materials

S2.1. Tensile Mechanical Characterization

The tensile mechanical properties of selected shielding materials, comprising the 0.5-mm lead reference, neat epoxy (NE), 50-phr Bi_2O_3–epoxy composite (B50), 50-phr WO_3–epoxy composite (W50), and hybrid multilayer B50/W50/B50/W50 composite (ML-BW50), were evaluated under uniaxial tensile loading. Dumbbell-shaped specimens were prepared and trimmed using a geometry based on the ISO 37 Type 2 configuration. Testing was performed using a ZwickRoell Z010 universal testing machine (ZwickRoell GmbH & Co. KG, Ulm, Germany) equipped with a 1 kN load cell. Each specimen was mounted with an initial gauge length of 20 mm. A preload of 0.1 N was applied before testing, followed by tensile loading at a crosshead speed of 100 mm·min^{-1} until specimen rupture. Force and extension were recorded continuously throughout each test.

Stress (σ) and strain (ε) were calculated as follows:

$$\sigma = \frac{F}{A_0} \quad \text{(S1)}$$

$$\varepsilon = \left(\frac{\Delta L}{L_0}\right) \times 100 \quad \text{(S2)}$$

Here, F is the applied tensile force, A_0 is the initial cross-sectional area of the reduced specimen section, ΔL is the change in gauge length, and L_0 is the initial gauge length. The resulting engineering stress–strain curves were used to determine the maximum force, maximum tensile stress, strain at maximum stress, and terminal strain corresponding to elongation at break.

S2.2. Tensile Stress–Strain Behavior and Derived Mechanical Properties

Figure S2 presents the tensile stress–strain behavior and derived mechanical parameters of the 0.5-mm lead reference, neat epoxy (NE), 50-phr Bi_2O_3–epoxy (B50), 50-phr WO_3–epoxy (W50), and four-layer B50/W50/B50/W50 composite (ML-BW50). The representative tensile stress–strain curves in Figure S2A demonstrate distinct mechanical responses among the materials. The lead reference sustained substantially higher tensile stress over a shorter strain range, whereas the epoxy-based specimens exhibited lower stress levels accompanied by markedly greater deformation. Among the epoxy formulations, NE and B50 extended over the greatest strain ranges, while W50 and ML-BW50 displayed similar curve termination regions. These profiles establish a clear strength–deformability contrast between the metallic reference and the polymer-based shielding materials.

The maximum force, Fmax, differed significantly among the groups according to Welch's one-way ANOVA [$F(4, 9.38) = 1328.82$, $p < 0.0001$; Figure S2B]. The lead reference exhibited the highest Fmax (58.74 ± 0.58 N), followed by NE (32.93 ± 2.58 N), B50 (27.63 ± 0.92 N), ML-BW50 (26.48 ± 2.44 N), and W50 (23.47 ± 1.26 N). Games–Howell comparisons with ML-BW50 showed significantly higher Fmax values for lead ($p < 0.0001$) and NE ($p = 0.022$). The differences between ML-BW50 and B50 (p = 0.853) and between ML-BW50 and W50 ($p = 0.222$) were not statistically significant. Therefore, the peak load sustained by ML-BW50 was within the measured range of the two high-loading monolithic composites, although it was lower than that of neat epoxy and the lead reference.

Maximum tensile stress, Tmax, also differed significantly among the groups [Welch's $F(4, 9.78) = 3390.64$, $p < 0.0001$; Figure S2C]. The lead reference exhibited a Tmax of 17.73 ± 0.20 MPa, substantially exceeding the values measured for NE (4.83 ± 0.85 MPa), B50

(3.74 ± 0.21 MPa), ML-BW50 (3.44 ± 0.30 MPa), and W50 (3.01 ± 0.30 MPa). In the comparisons focused on ML-BW50, only the lead reference exhibited a significant difference ($p < 0.0001$). No significant differences were detected between ML-BW50 and NE ($p = 0.085$), B50 ($p = 0.408$), or W50 ($p = 0.249$). Although NE had a numerically higher mean Tmax than ML-BW50, the difference did not reach statistical significance after adjustment for multiple comparisons. The filled epoxy formulations therefore occupied a similar maximum-stress range under the applied tensile-testing conditions.

Elongation at break showed a different ordering from the force- and stress-based parameters [Welch's $F(4, 9.91) = 114.27$, $p < 0.0001$; Figure S2D]. B50 exhibited the greatest elongation at break (91.59 ± 3.94%), followed by NE (83.80 ± 3.48%), W50 (67.38 ± 3.84%), ML-BW50 (64.55 ± 6.61%), and lead (30.42 ± 4.94%). Relative to ML-BW50, elongation was significantly lower for lead ($p = 0.0002$) and significantly higher for NE ($p = 0.0067$) and B50 ($p = 0.0009$). The difference between W50 and ML-BW50 was not statistically significant ($p = 0.913$). ML-BW50 consequently retained substantially greater deformation capacity than the lead reference, while its elongation response closely approached the mean measured for W50.

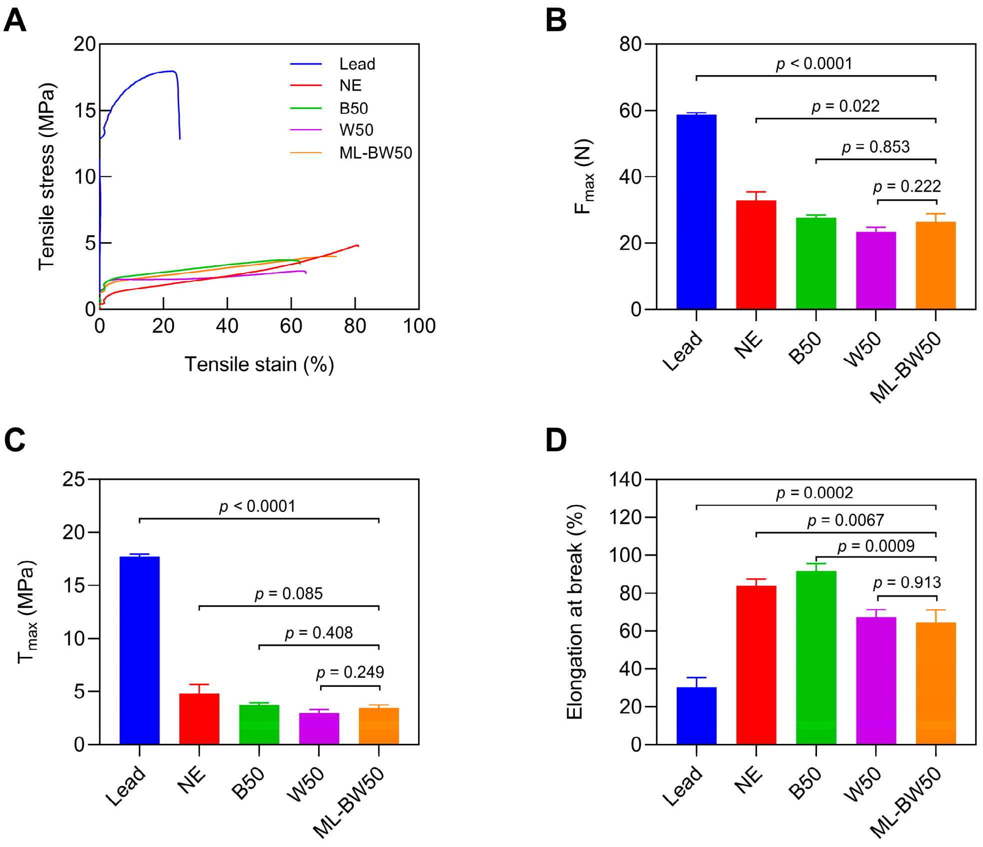

Figure S2. Tensile mechanical behavior of the 0.5-mm lead reference and selected epoxy-based shielding materials. (**A**) Representative engineering tensile stress–strain curves; (**B**) maximum force (Fmax); (**C**) maximum tensile stress (Tmax); and (**D**) elongation at break. Bars represent mean ± SD ($n = 5$ specimens). Overall group differences were evaluated using Welch's one-way ANOVA followed by Games–Howell multiple-comparison testing. Connecting brackets show multiplicity-adjusted comparisons between each material and ML-BW50; $p < 0.05$ was considered statistically significant. NE, the neat epoxy; B50, the epoxy containing 50 phr Bi_2O_3 nanoparticles; W50, the epoxy containing 50 phr WO_3 nanoparticles; ML-BW50, the four-layer B50/W50/B50/W50 hybrid multilayer composite.

Collectively, these results define the mechanical profile of ML-BW50 as moderate peak-load capacity, maximum tensile stress within the range of the monolithic nanoparticle-filled epoxy systems, and elongation markedly greater than that of the lead reference. Compared with NE, ML-BW50 exhibited numerical reductions of 19.6% in Fmax, 28.8% in Tmax, and 23.0% in elongation at break; the differences in Fmax and elongation were statistically significant, whereas the difference in Tmax was not. The nonparallel changes in force, stress, and elongation demonstrate that the effects of nanoparticle incorporation depend on filler composition and composite architecture. The lower peak force and elongation of ML-BW50 relative to NE are consistent with altered stress transfer and deformation within a matrix containing rigid particle-rich regions and multiple compositional interfaces. However, its similarity to B50 and W50 in Fmax and Tmax, together with its close correspondence to W50 in elongation, shows that the multilayer composite maintained the characteristic mechanical range of the high-loading nanoparticle-filled epoxy formulations evaluated in this study.

S3. Tensile Properties and Tensile Toughness

S3.1. Calculation Method

Tensile toughness was calculated as the mechanical energy absorbed per unit specimen volume during tensile loading. It corresponds to the area under the recorded engineering stress–strain curve from the initial measurement point to the final recorded stress value:

$$U_t = \int_0^{\epsilon t} \sigma(\varepsilon) d\varepsilon \quad \text{(S3)}$$

Here, U_t is tensile toughness, σ is tensile stress, ε is tensile strain, and ϵt is the terminal recorded strain used for integration.

Because the experimental data consisted of discrete stress–strain measurements, the integral was evaluated using the trapezoidal rule:

$$U_t = \sum \left\{ \left[\frac{(\sigma_i + \sigma_{i+1})}{2} \right] \right\} \times \left[\frac{(\varepsilon_{i+1} - \varepsilon_i)}{100} \right] \quad \text{(S4)}$$

Here, σ_i and σ_{i+1} are two consecutive tensile-stress measurements in MPa, and ε_i and ε_{i+1} are the corresponding tensile-strain measurements expressed as percentages. Division by 100 converts percentage strain into dimensionless strain. Because 1 Pa is equivalent to 1 $\mathrm{J \cdot m^{-}}$[illegible] integration of stress in MPa with respect to dimensionless strain gives tensile toughness directly in $\mathrm{MJ \cdot m^{-}}$[illegible](1 MPa = 1 $\mathrm{MJ \cdot m^{-}}$[illegible]).

For each material, integration commenced at the first recorded data point and terminated at the last nonblank stress measurement. Blank cells occurring after the end of a recorded stress–strain trace were excluded and were not assigned zero values. The measurements were integrated in their original recorded sequence without smoothing, interpolation, curve fitting, or extrapolation.

As an illustrative calculation, two consecutive ML-BW50 data points had the following values:

σ_i = 0.328050 MPa

σ_{i+1} = 0.350570 MPa

ε_i = 0.010518%

ε_{i+1} = 0.025543%

The contribution of this interval to the total tensile toughness was as follows:

ΔU_t = [(0.328050 + 0.350570)/2] × [(0.025543 − 0.010518)/100]

ΔU_t = 0.00005098 $\mathrm{MJ \cdot m^{-}}$[illegible]

The total tensile toughness was obtained by summing all individual trapezoidal contributions. For ML-BW50, integration through the final recorded stress value produced the following:

U_t,ML-BW50 = $\Sigma\Delta U_t$ = 2.249527 MJ·m^{-}◎

The value was reported to three decimal places as: U_t,ML-BW50 = 2.250 MJ·m^{-}◎

The relative difference in tensile toughness compared with neat epoxy was calculated as follows:

Difference vs. NE (%) = [(U_t,sample/U_t,NE) − 1] × 100

The percentage of the lead-reference value retained by each material was calculated as follows:

Retained vs. Pb (%) = (U_t,sample/U_t,Pb) × 100

For example, the relative difference between ML-BW50 and neat epoxy was as follows:

Difference vs. NE = [(2.249527/2.162693) − 1] × 100

Difference vs. NE = 4.015% ≈ 4.0%

The tensile toughness of ML-BW50 relative to the lead reference was as follows:

Retained vs. Pb = (2.249527/4.180294) × 100

Retained vs. Pb = 53.813% ≈ 53.8%

S3.2. Tensile Properties and Calculated Tensile Toughness

The calculated tensile-toughness values ranged from 1.571 to 4.180 MJ·m^{-}◎(Table S1). The 0.5-mm lead reference exhibited the greatest tensile toughness, 4.180 MJ·m^{-}◎, resulting from its substantially higher tensile stress over its recorded strain interval. Lead reached a maximum stress of 17.968 MPa at a strain of 22.502% and had a terminal recorded strain of 25.104%. Its high area under the stress–strain curve therefore resulted primarily from its elevated stress response, despite its lower terminal strain compared with the epoxy-based specimens.

Among the epoxy formulations, ML-BW50 exhibited the highest tensile toughness, 2.250 MJ·m^{-}◎. This value was 4.0% higher than that of neat epoxy, 16.7% higher than that of B50, and 43.2% higher than that of W50. ML-BW50 retained 53.8% of the tensile-toughness value measured for the lead reference. Additionally, neat epoxy exhibited the highest maximum stress and terminal strain among the epoxy-based specimens, reaching 4.826 MPa and 81.047%, respectively. Its tensile toughness was 2.163 MJ·m^{-}◎. Although the maximum stress and terminal strain of neat epoxy exceeded those of ML-BW50, the ML-BW50 trace maintained a higher stress response over a substantial portion of the intermediate and high-strain region. The integrated area under the complete ML-BW50 curve was consequently greater than that of neat epoxy. This result demonstrates that tensile toughness depends on the entire stress–strain response and cannot be determined from maximum stress or terminal strain alone.

The monolithic B50 composite produced a maximum stress of 3.721 MPa, a terminal strain of 62.816%, and a tensile toughness of 1.928 MJ·m^{-}◎. Its toughness was 10.8% lower than that of neat epoxy. W50 exhibited a slightly higher terminal strain than B50, at 64.656%, but its lower maximum stress of 2.898 MPa and lower stress response across much of the recorded strain interval resulted in the lowest tensile toughness, 1.571 MJ·m^{-}◎. Accordingly, B50 exhibited approximately 22.7% greater tensile toughness than W50.

The ML-BW50 composite reached a maximum tensile stress of 4.008 MPa at 72.050% strain and maintained measurable stress up to a terminal strain of 74.294%. Its comparatively broad stress–strain response produced the greatest integrated energy density among the epoxy formulations. The result indicates that the multilayer composite com-

bined a relatively high stress-bearing response with substantial tensile deformation, producing a more favorable balance of strength and deformation capacity than the corresponding B50 and W50 monolithic composites in the supplied tensile traces.

Overall, numerical integration of the stress–strain curves identified ML-BW50 as the toughest epoxy-based formulation, with a tensile toughness of 2.250 $MJ\cdot m^{-3}$, exceeding NE, B50, and W50 by 4.0%, 16.7%, and 43.2%, respectively. This performance resulted from the capacity of ML-BW50 to sustain comparatively high tensile stress across a broad strain interval, demonstrating a favorable balance between stress-bearing capacity and deformation. The tensile-toughness results therefore provide an integrated measure of energy absorption under uniaxial loading and complement the maximum-stress and terminal-strain measurements. The reported parameter is tensile toughness, expressed as energy absorbed per unit volume ($MJ\cdot m^{-3}$)

Table S1. Curve-derived tensile properties and tensile toughness of the 0.5-mm lead reference, neat epoxy, monolithic nanoparticle-filled composites, and the ML-BW50 hybrid multilayer composite.

**Material**	**Terminal strain (%)**	**Maximum stress (MPa)**	**Strain at maximum stress (%)**	**Tensile toughness[1] ($MJ\cdot m^{-3}$)**	**Difference vs. NE[2] (%)**	**Retained vs. Pb[3] (%)**
Lead	25.104	17.968	22.502	4.180	93.3	100.0
NE	81.047	4.826	80.815	2.163	0.0	51.7
B50	62.816	3.721	58.511	1.928	–10.8	46.1
W50	64.656	2.898	63.372	1.571	–27.3	37.6
ML-BW50	74.294	4.008	72.050	2.250	4.0	53.8

[1] Tensile toughness was calculated by the trapezoidal integration of the complete supplied stress–strain trace. [2] Difference vs. NE represents the percentage difference relative to neat epoxy. [3] Retained vs. Pb represents the tensile toughness expressed as a percentage of the 0.5-mm lead reference value.
